# Calcium Channel Blocker Lacidipine Promotes Antitumor Immunity by Reprogramming Tryptophan Metabolism

**DOI:** 10.1002/advs.202409310

**Published:** 2024-11-25

**Authors:** Yuwen Sheng, Chong Qiao, Zhonghui Zhang, Xiaoke Shi, Linhan Yang, Ruiying Xi, Jialing Yu, Wanli Liu, Guolin Zhang, Fei Wang

**Affiliations:** ^1^ Center for Natural Products Research Chengdu Institute of Biology Chinese Academy of Sciences Chengdu 610041 China; ^2^ School Of Pharmaceutical Sciences Sun Yat‐Sen University Guangzhou 511400 China; ^3^ University of Chinese Academy of Sciences Beijing 100049 China; ^4^ State Key Laboratory of Membrane Biology School of Life Sciences Institute for Immunology Beijing Advanced Innovation Center for Structural Biology Beijing Key Lab for Immunological Research on Chronic Diseases Beijing 100084 China

**Keywords:** breast cancer, calcium channel, indoleamine 2, 3‐dioxygenase, immunotherapy, lacidipine

## Abstract

Dysfunction of calcium channels is involved in the development and progression of some cancers. However, it remains unclear the role of calcium channel inhibitors in tumor immunomodulation. Here, calcium channel blocker lacidipine is identified to potently inhibit the enzymatic activity and expression of indoleamine 2,3‐dioxygenase 1 (IDO1), a rate‐limiting enzyme in tryptophan metabolism. Lacidipine activates effector T cells and incapacitates regulatory T cells (Tregs) to augment the anti‐tumor effect of chemotherapeutic agents in breast cancer by converting immunologically “cold” into “hot” tumors. Mechanistically, lacidipine targets calcium channels (Ca_V_1.2/1.3) to inhibit Pyk2‐JAK1‐calmodulin complex‐mediated IDO1 transcription suppression, which suppresses the kynurenine pathway and maintains the total nicotinamide adenine dinucleotide (NAD) pool by regulating NAD biosynthesis. These results reveal a new function of calcium channels in IDO1‐mediated tryptophan metabolism in tumor immunity and warrant further development of lacidipine for the metabolic immunotherapy in breast cancer.

## Graphical abstract

The antihypertensive drug lacidipine, as a bifunctional modulator of the enzymatic activity and expression of indoleamine 2,3‐dioxygenase 1 (IDO1), targets calcium channels to inhibit (Protein Tyrosine 2 (Pyk2)‐Janus Kinase 1 (JAK1)‐calmodulin complex‐mediated IDO1 immunosuppression. Lacidipine augments the anti‐tumor effect of chemotherapeutics by blocking the kynurenine pathway, maintaining the total nicotinamide adenine dinucleotide (NAD) pool and converting “cold” into “hot” tumors in triple‐negative breast cancer (TNBC).

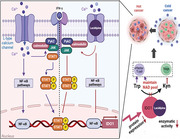

## 1 Introduction

Triple‐negative breast cancer (TNBC), a tumor subgroup poorly expressing Her2, the estrogen receptor, and the progesterone receptor, is the most lethal type of breast cancer with a higher metastatic potential, shorter patient survival, and higher rates of relapse.^[^ ^1^ ^]^ Moreover, because of the heterogeneity of TNBC, drug resistance and serious side effects are often observed, and clinical trials combining chemotherapy with targeted drugs have failed to show significant improvements in survival.^[^ ^2^ ^]^ Therefore, there is an urgent need to develop new treatment strategies. Tumors can be classified as “cold” or “hot” based on the level of tumor infiltrating lymphocytes (TILs), which is closely related to the prognosis and curability of the tumor. Increasing evidence has shown that the immune system is crucial to disease outcomes in TNBC and is strongly linked to improved survival.^[^ ^3^ ^]^ More importantly, TILs are independent prognostic biomarkers in breast cancer, and high TIL levels are associated with improved survival.^[^ ^4^ ^]^ Considering the importance of tumor immunosurveillance for cancer prevention and the high immunogenicity of TNBC, immunotherapy is becoming a promising and innovative treatment for TNBC. At present, a growing number of immunotherapeutic strategies are being developed for breast cancer, including vaccines, checkpoint inhibitors, and combination strategies; however, only atezolizumab in combination with nanoparticle albumin‐bound paclitaxel has been approved for immunotherapy in patients with unresectable locally advanced or metastatic TNBC. Therefore, it is in urgent need to develop new therapeutic strategies that convert immunologically “cold” into “hot” tumors to generate more effective anti‐tumor responses in TNBC.

The L‐type calcium channels (Ca_V_1 channels), belonging to voltage gated calcium channels, have been found to be closely associated with the initiation and progression of cancer.^[^ ^5^, ^6^ ^]^ Compared with healthy counterparts, cancer cells have a depolarized cell membrane potential, which may facilitate abnormal activation of Ca_V_1 channels.^[^ ^7^ ^]^ Excessive activation of Ca_V_1 channels and changes in intracellular Ca^2+^ concentration also lead to the proliferation, invasion, and metastasis of tumor cells.^[^ ^6^, ^8^ ^]^ Dihydropyridines (e.g., nimodipine, nifedipine, amlodipine, lacidipine) are a type of L‐type calcium channel blockers (CCBs) mainly used in the clinical treatment of hypertension, which interact with Ca_V_1 channels to block the depolarization of vascular smooth muscle cells, myocardial cells, and heart node tissue to inhibit Ca^2+^ influx, thereby slowing down the heart rate, reducing myocardial contractile function, and eventually lowering blood pressure.^[^ ^6^ ^]^ Nifedipine has been extensively studied for its anti‐tumor effects, and previous studies have shown that nifedipine exerts anti‐tumor immunomodulatory effect by inhibiting calcium influx to impair nuclear factor of activated T cell 2 (NFAT2) and reducing the expression of programmed death ligand 1 (PD‐L1) on colorectal cancer cells and programmed death ligand 1 (PD‐1) on CD8^+^ T cells to reactivate tumor immune surveillance.^[^ ^9^, ^10^ ^]^ However, some studies suggested that dihydropyridines exert the anti‐tumor effects not by blocking calcium channels, such as amlodipine.^[^ ^11^ ^]^ Lacidipne, a novel third‐generation dihydropyridine CCBs, has a more potent and long‐lasting antihypertensive effect than nifedipine.^[^ ^12^ ^]^ Lacidipine has previously been found to inhibit the viability and proliferation of ovarian tumor stem cells by the induction of apoptosis.^[^ ^13^ ^]^ Therefore, further investigation of CCBs such as lacidipine will be important to elucidate the role of Ca_V_1 channels and the therapeutic potential of CCBs in tumor immunomodulation.

In humans, tryptophan (Trp) levels depend on food intake and the activity of several Trp metabolic pathways, including kynurenine (Kyn), 5‐hydroxytrptamine (HT), and indole pathways. Among these pathways, Kyn is the major Trp metabolic pathway, and > 95% Trp degrades into multiple bioactive compounds through this pathway.^[^ ^14^ ^]^ Indoleamine‐2,3‐dioxygenase 1 (IDO1), IDO2, and Trp‐2,3‐dioxygenase (TDO) are crucial rate‐limiting enzymes in the Kyn pathway. These enzymes degrade Trp, resulting in the accumulation of kyn metabolites, which promote the differentiation of regulatory T cells (Tregs) and inhibit the activity of effector T cells. This process contributes to local immunosuppression within the tumor microenvironment, facilitating the survival and escape of cancer cells.^[^ ^15^ ^]^ IDO1 expression is typically induced by pro‐inflammatory cytokines such as interferon γ (IFN γ) and tumor necrosis factor‐α (TNF‐α) and is regulated by JAK/STAT and NF‐κB signaling pathways;^[^ ^16^ ^]^ However, the mechanisms by which these pro‐inflammatory cytokines interact with other signaling pathways to collectively modulate the homeostasis of tryptophan metabolism remain unclear. Accumulating evidences have demonstrated that intracellular Ca^2+^ homeostasis is altered in cancer cells, which is implicated in the regulation of JAK/STAT or NF‐κB signaling pathways.^[^ ^17^, ^18^ ^]^ Moreover, tryptophan and Ca^2+^ ions cooperatively activate the human calcium‐sensing receptor, and L‐type calcium channels can modulate tryptophan hydroxylase in the rat pineal gland.^[^ ^19^, ^20^ ^]^ However, it remains unknown whether calcium channels modulate tryptophan metabolism and blocking them can benefit in cancer immunotherapy.

Several studies have shown high IDO1 expression was associated with poor prognosis in a variety of cancer types.^[^ ^21^ ^]^ Undoubtedly, IDO1 is an ideal target for cancer immunotherapy, several IDO1 inhibitors, including 1‐methyl‐D, L‐tryptophan, and indoximod, have entered clinical trials. However, IDO1 inhibitors alone have not shown satisfactory therapeutic effects and need to be combined with chemotherapy and immunotherapy. In addition, the failure of a phase III trial of epacadostat in combination with pembrolizumab for the treatment of melanoma revealed deficiencies in existing IDO1 inhibitor treatment strategies, which may be caused by inadequate intratumor‐specific IDO1 inhibition, compensatory immune escape through tumor production of TDO, activation of downstream effector pathways such as the aryl hydrocarbon receptor (AHR), suboptimal combination therapy strategies, and excessive nicotinamide adenine dinucleotide (NAD)‐mediated T cell inactivation.^[^ ^22^, ^23^ ^]^ Notably, preclinical mouse tumor models suggest that IDO1‐mediated immunosuppression is not merely dependent on its enzymatic activity^[^ ^24^ ^]^ and that IDO1 may act as a signaling molecule to mediate or maintain immune tolerance independent of its enzymatic activity. Therefore, it may be necessary to simultaneously target the transcriptional level of IDO and inhibit its enzymatic activity to effectively reverse IDO‐mediated immunosuppression. In this study, through high‐throughput screening of Kyn production in cancer cells using an FDA‐approved drug library, we found that lacidipine significantly inhibited kynurenine synthesis not only by suppressing the expression of IFN γ‐induced IDO1/2 and TDO2, but also by inhibiting the catalytic activity of IDO1. Lacidipine potently augmented the anti‐tumor effects of chemotherapeutic agents on breast cancer in vivo by activating CD45^+^CD8^+^ effector T cells and incapacitating CD4^+^CD25^+^Foxp3^+^ Tregs. Furthermore, lacidipine effectively blocked the kynurenine pathway of tryptophan degradation, meanwhile promoting a metabolic adaptation that preserved the total NAD pool through the regulation of various NAD biosynthesis pathways. In addition, lacidipine regulated the expression of “cold” and “hot” tumor‐related genes and transformed “cold” into “hot” tumors, providing evidence for its potent immunomodulation‐enhancing effects in vitro and in vivo. We further demonstrated that lacidipine inhibited the formation of the calcium‐mediated Pyk2‐JAK1‐calmodulin complex, thereby inhibiting the JAK/STAT and NF‐κB signaling pathways in IDO1 expression. These results reveal a new function of calcium channels in IDO1‐mediated tryptophan metabolism in tumor immunity and warrant further development of lacidipine as a safer and more effective metabolic immunotherapy for breast cancer.

## 2 Results

### 2.1 Lacidipine Attenuates the Enzymatic Activity and Expression of IDO1

**Figure 1:**
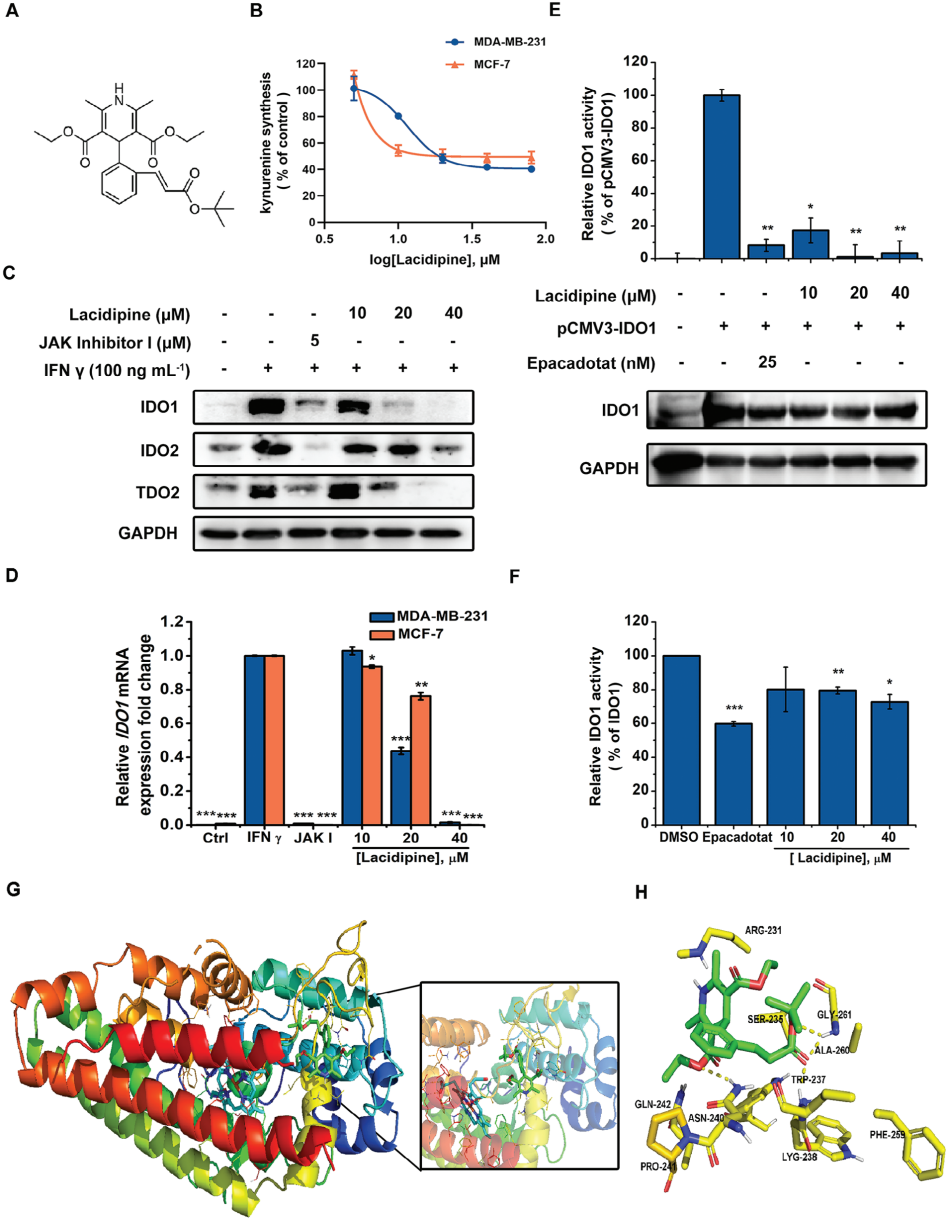
Lacidipine attenuates the enzymatic activity and expression of IDO1. A) Chemical structure of lacidipine. B) MDA‐MB‐231 and MCF‐7 cells were treated with lacidipine (5 × 10^−6^, 10 × 10^−6^, 20 × 10^−6^, 40 × 10^−6^, and 80 × 10^−6^ m) for 2 h, and then treated with IFN γ (100 ng mL^−1^) for 24 h. The concentration of kynurenine in the cell supernatants was determined. Bars, ± standard error of mean (SEM). The curves were plotted using a variable slope (four‐parameter) non‐linear fit. C) Effect of lacidipine on the IDO1 expression. MDA‐MB‐231 cells were pre‐treated with lacidipine (10 × 10^−6^, 20 × 10^−6^, and 40 × 10^−6^ m) and JAK inhibitor I (5 × 10^−6^ m) for 2 h, then stimulated with IFN γ (100 ng mL^−1^) for 24 h. The expression of IDO1, IDO2, and TDO2 was analyzed by western blotting. GAPDH was used as the loading control. D) MDA‐MB‐231 and MCF‐7 cells were pre‐treated with lacidipine (10 × 10^−6^, 20 × 10^−6^, and 40 × 10^−6^ m) and JAK inhibitor I (5 × 10^−6^ m) for 2 h, then stimulated with IFN γ (100 ng mL^−1^) for 24 h. The mRNA expressions of IDO1 examined by qRT‐PCR. The mRNA levels of these genes were normalized against GAPDH expression levels. Bars, ± SEM. *p < 0.05, **p < 0.01, and ***p < 0.001 versus IFN γ treatment group (unpaired two‐tailed Student's *t*‐test). E) HEK293A cells are transfected with pCMV3‐IDO1 plasmids for 48 h, and then treated with lacidipine (10 × 10^−6^, 20 × 10^−6^, and 40 × 10^−6^ Μ) and epacadostat (25 × 10^−9^ m) for 6 h. Cell supernatants were used to detect the activity of IDO1. Bars, ± SEM. *p < 0.05, **p < 0.01 versus pCMV3‐IDO1 group (unpaired two‐tailed Student's *t*‐test). The cell lysates were immunoblotted with IDO1 antibody. GAPDH was used as the loading control. All experiments were conducted with three independent replicates. F) The catalytic activity of the recombinant expressed IDO1 protein was measured. G) The binding location of lacidipine in the IDO1 protein. H) Detailed interactions between lacidipine and the active binding site of the IDO1 protein.

Through a high‐throughput screening against Kyn production induced by IFN γ in HeLa cells using an FDA‐approved drug library, we identified that lacidipine is a potent inhibitor of Kyn production. The chemical structure of lacidipine is shown in **Figure** **1**A. Lacidipine was found to inhibit the IFN γ‐induced Kyn production in MDA‐MB‐231, MCF‐7, and HeLa cells in a dose‐dependent manner, with IC_50_ values of 12.32 ± 1.20 × 10^−6^, 13.57 ± 0.45 × 10^−6^ and 16.81 ± 2.49 × 10^−6^ m, respectively (Figure 1B and Figure S1A, Supporting Information), without significant cytotoxic effect in these cells (Figure S1A,B, Supporting Information). We evaluated the effect of lacidipine on the expression of IDO1, IDO2, and TDO2, which play key roles in the catalytic conversion of Trp to Kyn. As shown Figure 1C and Figure S1C (Supporting Information), lacidipine remarkably decreased the expression of IDO1, IDO2, and TDO2 in MDA‐MB‐231, MCF‐7, and 4T1 cells and inhibited the IFN γ‐induced upregulation of IDO1 mRNA expression in MDA‐MB‐231 and MCF‐7 cells (Figure 1D). To further validate the inhibitory effect of lacidipine on IDO1/IDO2/TDO2 enzyme activity, we overexpressed IDO1, IDO2, and TDO2 in HEK293A cells. We found that lacidipine inhibited IDO1/2 enzymatic activity but had no effect on TDO2 activity (Figure 1E and Figure S1D,E, Supporting Information). Consistent with this, lacidipine significantly inhibited the enzymatic activity of recombinant expressed IDO1 (Figure 1F). In addition, we performed a cellular thermal shift assay (CETSA), a method for evaluating intracellular drug–protein interactions,^[^ ^25^ ^]^ to elucidate the potential interactions between lacidipine and IDO1 proteins. Compared to the DMSO group, lacidipine promoted the accumulation of IDO1 protein in cell lysates with an increase in temperature and concentrations of lacidipine (Figure S1F,G, Supporting Information). Molecular docking was performed to examine the mode of interaction between lacidipine and IDO1 (Figure 1G,H). Lacidipine was embedded in the IDO1 active pocket by forming four hydrogen bonds with the amino acid residues TRP‐237, ASN‐240, ALA‐260, and GLY‐261 to maintain the stability of the combination. Hydrophobic forces were formed by ARG‐231, SER‐235, GLN‐242, PRO‐241, LYG‐238, and PHE‐259 residues.

Next, we evaluated whether other dihydropyridine antihypertensive drugs have an effect similar to that of lacidipine. The IC_50_ values of isradipine, nifedipine, and nitrendipine on inhibiting Kyn biosynthesis were 23.33 ± 1.87 × 10^−6^ m, 26.74 ± 1.06 × 10^−6^ m, and >80 × 10^−6^ m, respectively, and the potency was lower than that of lacidipine (Figure S1H, Supporting Information). Moreover, the inhibitory effect of nifedipine on IDO1 activity may be partially attributed to its cytotoxic effects (Figure S1H, Supporting Information). In HEK293A cells overexpressing IDO1 or IDO2, these drugs also inhibited the enzymatic activity of IDO1 and IDO2 (Figure S1I–L, Supporting Information), although they slightly inhibited TDO2 (Figure S1M, Supporting Information). In addition, isradipine inhibited the expression of IDO1/IDO2/TDO2 (Figure S1N, Supporting Information), indicating that the dihydropyridine antihypertensive drugs had similar inhibition efficiencies on tryptophan metabolism, whereas lacidipine exhibited the highest potency. Taken together, these results suggest that lacidipine suppressed the IFN γ induced expression of IDO1/2 and TDO2 and inhibited enzymatic activity of IDO1/2, leading to efficient blockade of the Trp degradation via the Kyn pathway.

### 2.2 Lacidipine Exerts Potential Antitumor Effects In Vivo

**Figure 2:**
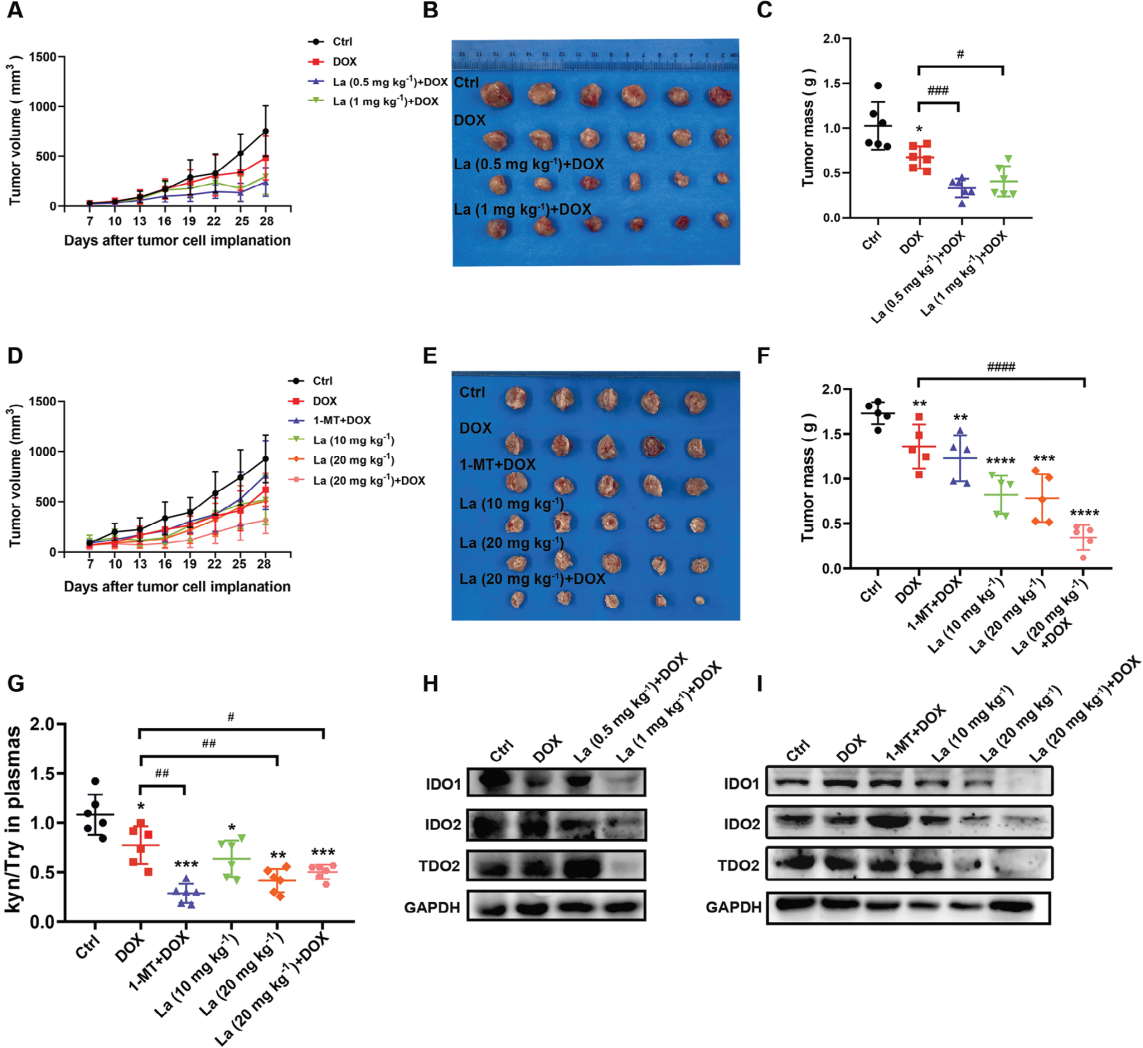
Lacidipine exerts potential anti‐tumor effects in vivo. A) Tumor volumes were recorded in the DOX only, and DOX + lacidipine (0.5 or 1 mg kg^−1^) groups (*n* = 6, each group) and are presented as the mean ± SEM. B) Image of the tumors in the DOX only, and DOX + lacidipine (0.5 or 1 mg kg^−1^) groups (*n* = 6, each group). C) Tumor mass was recorded in the DOX only, and DOX + lacidipine (0.5 or 1 mg kg^−1^) groups (*n* = 6, each group) and the results are presented as the mean ± SEM; *p < 0.05 versus control group and #*p* < 0.05, ###*p* < 0.001 versus DOX‐treated group (unpaired two‐tailed Student's *t*‐test). D) Tumor volumes were recorded in the DOX only, lacidipine (10 or 20 mg kg^−1^) only, and DOX + lacidipine (20 mg kg^−1^)/1‐MT groups (*n* = 12, each group) and are presented as the mean ± SEM. E) Image of the tumors in the DOX only, lacidipine (10 or 20 mg kg^−1^) only, and DOX + lacidipine (20 mg kg^−1^)/1‐MT groups (*n* = 6, each group). F) Tumor mass was recorded in the DOX only, lacidipine (10 or 20 mg kg^−1^) only, and DOX + lacidipine (20 mg kg^−1^)/1‐MT groups (*n* = 6, each group) and the results are presented as the mean ± SEM; **p < 0.01, ***p < 0.001, and ****p < 0.0001 versus control group and ####*p* < 0.0001 versus DOX‐treated group (unpaired two‐tailed Student's *t*‐test). G) The ratio of Kyn/Try in mouse serum was determined by HPLC at the end of the treatment. *p < 0.05, **p < 0.01 and ***p < 0.001 versus control group and #*p* < 0.05, ##*p* < 0.01 versus DOX‐treated group (unpaired two‐tailed Student's *t*‐test). H,I) The expression of IDO1, IDO2 and TDO2 were analyzed by western blotting in mouse tumor tissue homogenate of at the end of treatment. GAPDH was used as the loading control. The experiments were conducted with three independent replicates.

To evaluate the anti‐tumor activity of lacidipine in vivo, we established 4T1‐Luc breast tumor‐bearing BALB/c mice. After 7 d, the mice were divided into several groups that were administered lacidipine alone or in combination with the antineoplastic drugs doxorubicin (DOX) or cisplatin. The body weight of the mice was monitored every 3 d to evaluate the safety of the drug. Compared to the control group, treatment with the chemotherapeutic agents DOX or cisplatin alone resulted in body weight loss in the mice; however, lacidipine administration did not affect the body weight of the mice. In addition, the effect of 1‐MT or lacidipine in combination with DOX/cisplatin was similar to that in mice treated with DOX/cisplatin alone, indicating that the systemic toxicity of the combined administration was due to the toxicity of DOX/cisplatin, and lacidipine had little systemic toxicity in mice (Figure S2A–C, Supporting Information). Compared to the DOX group, lacidipine administered at 0.5 mg kg^−1^ (approximately 0.825 times the clinical dose of 4 mg day^−1^) and 1 mg kg^−1^ (approximately 1.65 times the clinical dose and 0.825 times the maximum clinical dose of 8 mg day^−1^) significantly inhibited tumor growth when combined with DOX (**Figure** **2**A–C). To further evaluate the safety and efficacy of lacidipine in vivo, we used high doses of lacidipine (10 and 20 mg kg^−1^, p.o.), as previously reported that amlodipine (10 mg kg^−1^, i.p.), a calcium channel inhibitor, significantly inhibited tumor growth in human epidermoid carcinoma with certain safety in vivo.^[^ ^11^ ^]^ Lacidipine (10 or 20 mg kg^−1^) significantly inhibited the growth rate of the tumor, and co‐administration with DOX/cisplatin showed potent therapeutic efficacy, superior to 1‐MT combined with DOX/cisplatin or DOX/cisplatin alone (Figure 2D–F and Figure S2D–F, Supporting Information). Live imaging was performed to detect the luminescence intensity of tumor growth in mice bearing 4T1‐Luc cells on days 7, 16, and 28. The results showed that all treatment groups had significantly reduced areas of luminescence compared with the control group, and the combination of lacidipine with DOX or cisplatin demonstrated superior inhibition of luminescence intensity in tumor growth compared to DOX or cisplatin treatment alone (Figure S2G, Supporting Information). To further verify the effect of lacidipine on IDO activity in vivo, Kyn/Try ratio in mouse serum was determined by high performance liquid chromatography (HPLC). Lacidipine treatment significantly reduced the Kyn/Trp ratio. Importantly, the co‐administration of lacidipine with DOX/cisplatin potently inhibited the Kyn/Trp ratio compared to DOX/cisplatin alone (Figure 2G and Figure S2H, Supporting Information). In addition, lacidipine (0.5 and 1 mg kg^−1^) reduced the expression of IDO1/2 in tumor tissues (Figure 2H and Figure S2I, Supporting Information). The administration of lacidipine alone and in combination with DOX/cisplatin also decreased the expression of IDO1/2 and TDO2 with greater efficacy than 1‐MT plus DOX/cisplatin co‐treatment (Figure 2I and Figure S2J,K, Supporting Information). These results suggest that lacidipine exerts preferable anti‐breast cancer effects in vivo by inhibiting IDO1 in combination with chemotherapeutic agents even at human clinical used safe dosage.

### 2.3 Lacidipine Exerts Immunomodulatory Effects In Vivo

**Figure 3:**
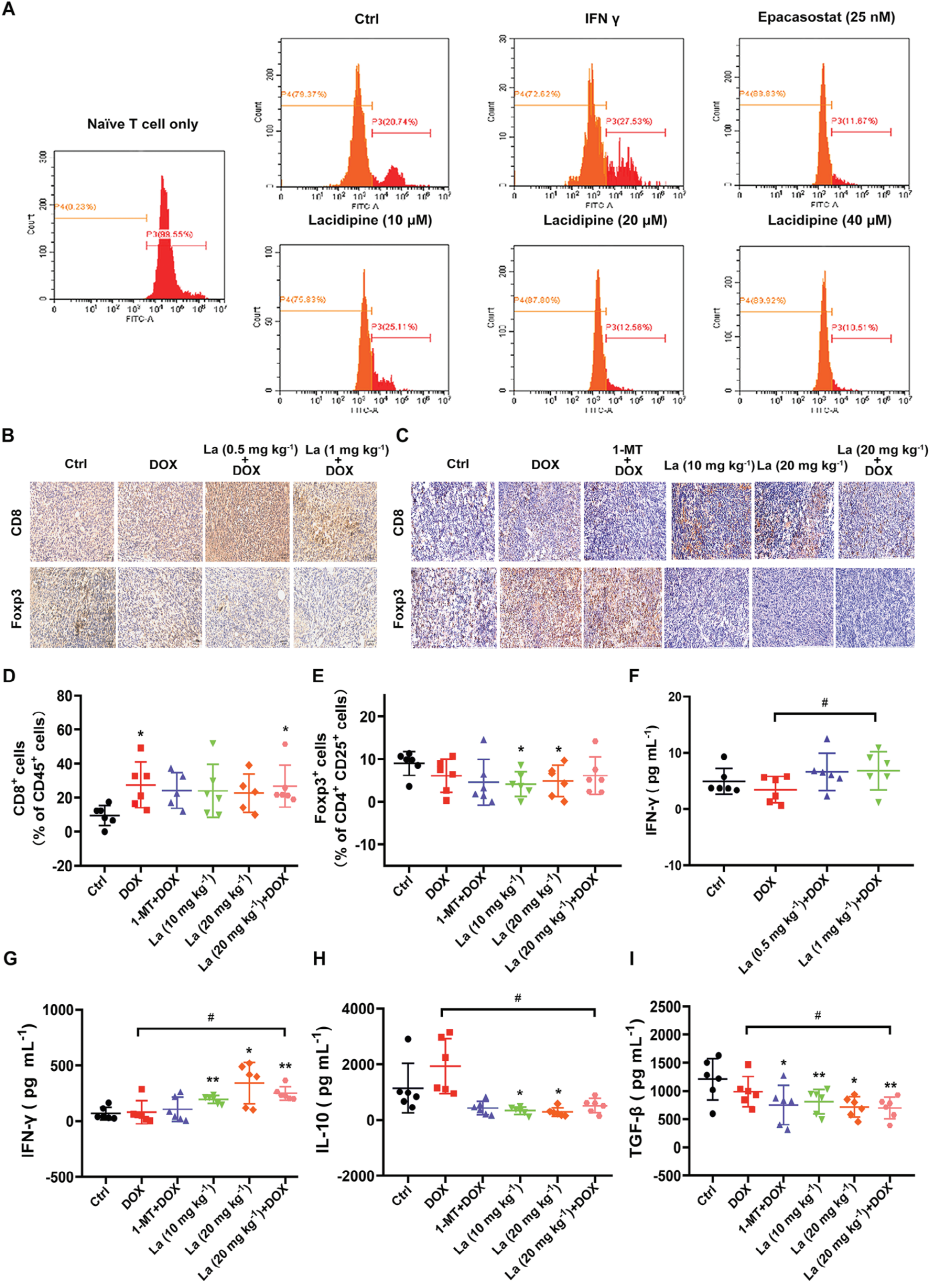
Lacidipine exerts immunomodulatory effects in vivo. A) Effect of lacidipine on the proliferation of T cells co‐cultured with IFN γ‐treated MDA‐MB‐231. The proliferation of CD8^+^ T cells was detected based on CFSE using flow cytometry (FACS). The percentage of proliferating cells is shown. The experiments were conducted with three independent replicates. B,C) The expression of CD8 and Foxp3 were examined by immunohistochemical analysis in tumor tissues (*n* = 6, each group). Representative images are shown. Scale bar = 250 µm. D) Percentage of CD8^+^ effector T cells in CD45^+^TILs in tumors was analyzed using flow cytometry at the end of treatment (*n* = 6, each group) and the results are presented as the mean ± SEM; *p < 0.05 versus control group (unpaired two‐tailed Student's *t*‐test). E) Percentage of CD4^+^CD25^+^Foxp3^+^ regulatory T cells in tumors was analyzed using flow cytometry at the end of treatment (*n* = 6, each group) and the results are presented as the mean ± SEM; *p < 0.05 versus control group (unpaired two‐tailed Student's *t*‐test). F–I) The levels of cytokines IFN γ, IL‐10, and TGF‐β were evaluated in tumor tissue homogenates by ELISA (*n* = 6, each group). *p < 0.05 and **p < 0.01 versus control group and #*p* < 0.05 versus DOX‐treated group (unpaired two‐tailed Student's *t*‐test). La, Lacidipine.

To evaluate the potential effect of lacidipine on CD8^+^ T cell proliferation, we co‐cultured MDA‐MB‐231, MCF‐7, and 4T1 cells with CD8^+^ T cells extracted from the mouse spleen in vitro. Compared with the findings in the IFN γ group, lacidipine administration promoted the proliferation of CD8^+^ T cells co‐cultured with MDA‐MB‐231, MCF‐7, and 4T1 cells (**Figure** **3**A and Figure S3A,B, Supporting Information). We then examined the immunomodulatory effect of lacidipine on T cells infiltration in the tumor tissue. Immunohistochemical analysis showed that lacidipine (0.5, 1, and 20 mg kg^−1^) in combination with DOX/cisplatin increased CD8 expression and decreased Foxp3 expression in tumor tissues (Figure 3B,C and Figure S3C, Supporting Information). Consistently, lacidipine increased the infiltration of CD45^+^CD8^+^ T cells in tumors, and lacidipine (20 mg kg^−1^) combined with DOX/cisplatin further increased the percentage of CD45^+^CD8^+^ T cells compared to the control group (Figure 3D and Figure S3D–F, Supporting Information). Lacidipine also significantly inhibited tumor‐infiltrating CD4^+^CD25^+^Foxp3^+^ Tregs. Furthermore, compared to cisplatin treatment alone, the combination of lacidipine with cisplatin markedly reduced the number of CD4^+^CD25^+^Foxp3^+^ Tregs. (Figure 3E and Figure S3G–I, Supporting Information). Next, we assessed the function of infiltrating effector T cells and Tregs in tumor tissues by detecting the levels of cytokines IFN γ, IL‐10, and TGF‐β in homogenates of the tumor tissues by enzyme‐linked immunosorbent assay (ELISA). Compared to DOX/cisplatin, the co‐administration of lacidipine (1 and 20 mg kg^−1^) and DOX/cisplatin also clearly increased the level of IFN γ (Figure 3F,G and Figure S3L, Supporting Information). In addition, lacidipine (20 mg kg^−1^) in combination with DOX notably decreased the levels of IL‐10 and TGF‐β compared to DOX alone (Figure 3H,I). Lacidipine alone also significantly promoted the level of IFN γ and inhibited the levels of IL‐10 and TGF‐β (Figure 3G–I and Figure S3L–N, Supporting Information). These results indicate that lacidipine accelerated the infiltration, and improved the function of effector T cells in tumor tissues, and incapacitated Tregs, thereby mediating tumor immunity and exerting anti‐tumor activity.

### 2.4 Lacidipine Blocks the Kyn Pathway and Maintains the Total NAD Pool

**Figure 4:**
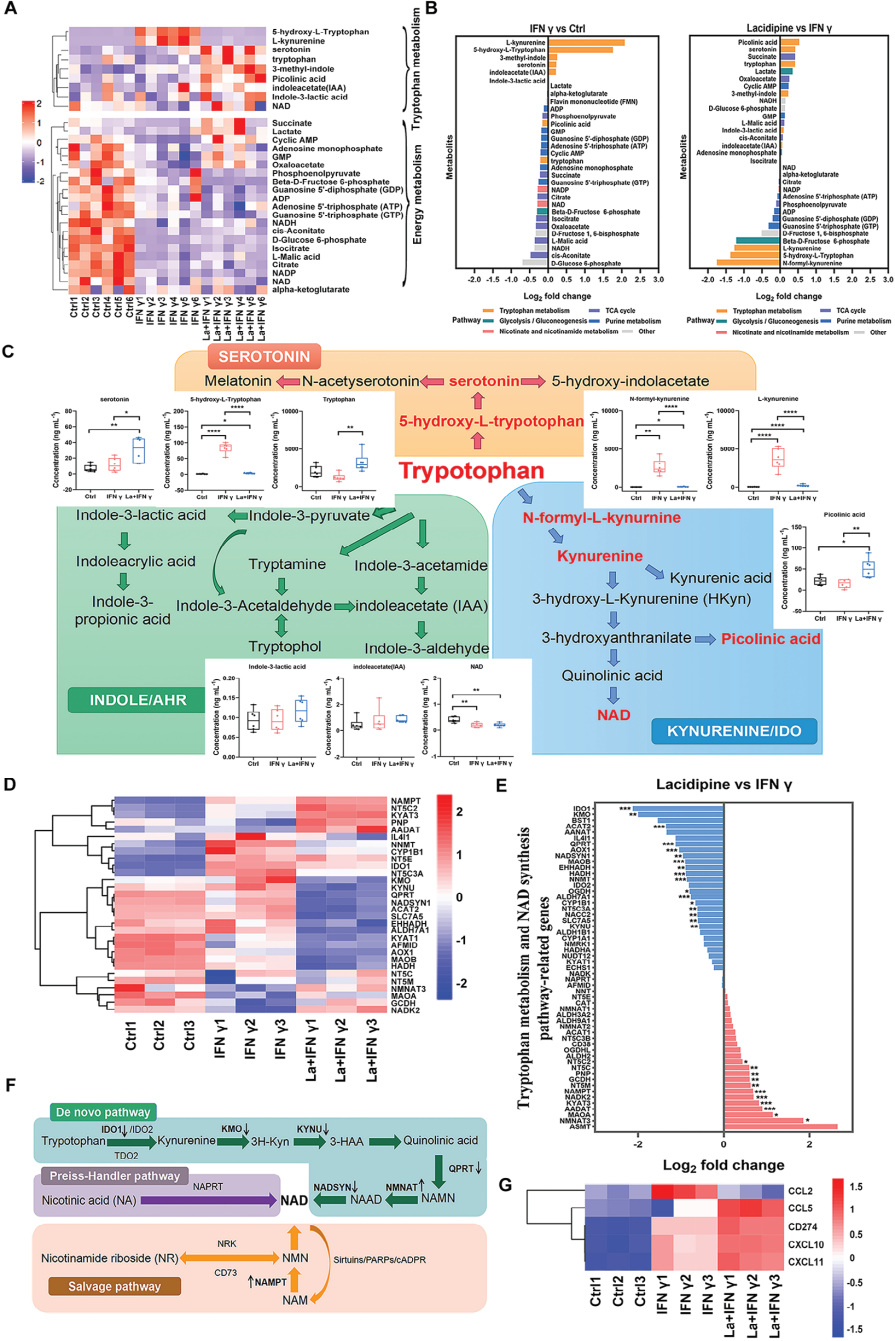
Lacidipine blocks the Kyn pathway and maintains the total NAD pool. A) Heatmap of the major metabolites in Trp and energy metabolic pathways based on the results of metabolome analysis (*n* = 6, each group). B) The alterations of key metabolite classes and abundance was based on IFN γ versus control and lacidipine versus IFN γ (*n* = 6, each group). C) Schematic summary of lacidipine‐induced changes in the Trp metabolic pathway. D) Heat map hierarchical clustering displays differentially expressed genes of tryptophan metabolism and the NAD synthesis pathway with *p* < 0.05 (*n* = 3, each group). E) The transcript log2 fold changes (x axis) of Trp catabolism and NAD synthesis pathway based on lacidipine versus IFN γ. *p < 0.05, **p < 0.01, and ***p < 0.001 versus IFN γ‐treated group (unpaired two‐tailed Student's *t*‐test). F) Schematic summary of lacidipine‐induced gene changes in the NAD synthesis pathway. G) Heat map hierarchical clustering displays the expression of “hot” tumor‐related genes (*CXCL10*, *CXCL11*, *CD274* and *CCL5*) and “cold” tumor‐related gene (*CCL2*) with a *p* < 0.05 (*n* = 3, each group). La, Lacidipine.

One potential mechanism for the failure of the IDO1 inhibitor epacadostat in clinical trials is that IDO1 inhibition leads to an effective blockade of the Kyn pathway and is accompanied by metabolic adaptation, which promotes the NAD biosynthesis pathway and inhibits T cell proliferation and function.^[^ ^23^ ^]^ To further explore the regulatory role of lacidipine‐induced downstream adaptive metabolism in tumor immunity, we investigated the effects of lacidipine on key metabolites in the Trp and energy metabolism pathways using metabolomic analysis (**Figure** **4**A and Figure S4A, Supporting Information). As expected, lacidipine significantly inhibited the increase in intermediate metabolites in the IFN γ‐mediated Trp metabolism pathway, including N‐formyl‐L‐kynurenine, L‐kynurenine, and 5‐hydroxy‐L‐tryptophan, which was accompanied by intracellular accumulation of Trp (Figure 4A,C). In addition, IFN γ regulated the related metabolites in the TCA cycle, glycolysis/gluconeogenesis, purine metabolism, and nicotinate and nicotinamide metabolic pathways (Figure 4B). Compared to IFN γ, lacidipine elevated intracellular levels of oxaloacetate (OAA) in the TCA cycle, as well as cyclic AMP and GMP in the purine metabolic pathway, while decreasing GDP and GTP levels. Moreover, it inhibited the production of β‐D‐fructose 6‐phosphate in the glycolysis/gluconeogenesis pathway (Figure 4B and Figure S4A, Supporting Information). Interestingly, lacidipine had no effect on the IFN γ‐mediated inhibition of NAD and metabolites in the Trp‐indole metabolic pathway (Figure 4B,C). The lacidipine‐induced metabolite changes in the Trp metabolic pathway are illustrated in Figure 4C.

To further validate the adaptive metabolic regulation of lacidipine upon IDO blockade, we performed transcriptomic analysis. Consistent with the metabolomic analysis, transcriptomics revealed that lacidipine regulated the expression of genes involved in IFN γ‐mediated Trp metabolism and NAD biosynthesis (Figure 4D). However, lacidipine downregulated kynurenine monooxygenase (*KMO*) and Kynureninase (*KYNU*), suggesting that lacidipine altered other downstream enzymatic steps of Kyn production (Figure 4E). The three main pathways of NAD biosynthesis are as follows: the de novo pathway (Kyn pathway), the Preiss–Handler pathway, and the salvage pathway. We observed that lacidipine modulated the de novo pathway, as evidenced by the downregulation of NAD synthetase (*NADSYN*) and quinolinate phosphoribosyltransferase (*QPRT*), the enzyme responsible for catalyzing the conversion of quinolinic acid (QA) to nicotinic acid mononucleotide (NAMN). Additionally, lacidipine increased the expression of nicotinamide mononucleotide adenylyl transferase (*NMNAT*), nicotinamide phosphoribosyltransferase (*NAMPT*), and maintained the expression of nicotinic acid phosphoribosyltransferase (*NAPRT*), an enzyme responsible for converting nicotinic acid (NA) to NAMN (Figure 4E). Furthermore, lacidipine inhibited the expression of solute carrier family 7 member 5 (*SLC7A5*), a 3‐hydroxykynurnine (3H‐Kyn), and the Trp transmembrane reverse transporter, which inhibited NAD resynthesis by decreasing the ratio of extracellular to intracellular Trp and increasing the ratio of extracellular to intracellular 3H‐Kyn (Figure 4E).^[^ ^26^ ^]^ Lacidipine‐induced gene changes in the NAD biosynthesis pathway are shown in Figure 4F. Lacidipine also regulated the expression of genes related to energy metabolism (Figure S4B, Supporting Information). Moreover, our transcriptomic data further revealed that lacidipine regulated multiple immune‐related genes, significantly upregulating “hot” tumor‐related genes such as *CCL5*, *CD274*, *CXCL10*, and *CXCL11*, while downregulating the “cold” tumor‐related gene *CCL2* (Figure 4G). Subsequently, we examined the mRNA levels of *CCL5*, *CD274*, *CXCL10*, *CXCL11*, and *CCL*2 in MDA‐MB‐231 and 4T1 cells and tumor tissues. The results showed that lacidipine significantly upregulated *CCL5* and *CXCL10* and decreased *CCL2* mRNA expression in MDA‐MB‐231 and 4T1 cells but inhibited *CXCL11* mRNA expression in MDA‐MB‐231 cells (Figure S4C, Supporting Information). Consistently, lacidipine treatment or co‐administration with DOX/cisplatin significantly increased the mRNA expression of *CCL5*, *CD274*, *CXCL10*, and *CXCL11* and decreased *CCL2* mRNA levels in tumor tissues (Figure S4D–H, Supporting Information), thereby promoting immune cell infiltration and converting the “cold” into a “hot” tumor. PD‐L1, as an immune checkpoint molecule, is often employed by tumor cells as a defense mechanism in “hot” tumors. When T cells recognize and attack these tumor cells, the tumor cells upregulate PD‐L1 expression to suppress T cell activity.^[^ ^27^, ^28^ ^]^ To further investigate, we examined PD‐L1 expression in the breast cancer cell lines MDA‐MB‐231, MCF‐7, and 4T1. The results revealed that PD‐L1 expression was lowest in MDA‐MB‐231 cells and highest in 4T1 cells (Figure S4I, Supporting Information). Moreover, lacidipine significantly suppressed PD‐L1 expression in MDA‐MB‐231, MCF‐7, and 4T1 cells (Figure S4J–L, Supporting Information). Collectively, lacidipine effectively blocked the Kyn pathway and reduced the levels of metabolites and expression of genes involved in the Trp metabolic pathway. Simultaneously, lacidipine maintained the total NAD pool by regulating the genes related to the NAD biosynthesis pathway and transformed “cold” into “hot” tumors by regulating the gene expression of “cold” and “hot” tumors, thereby exhibiting a positive immunomodulatory effect.

### 2.5 Lacidipine Inhibits the IDO1 Expression through Suppression of JAK/STAT and NF‐κB Signaling Pathways

**Figure 5:**
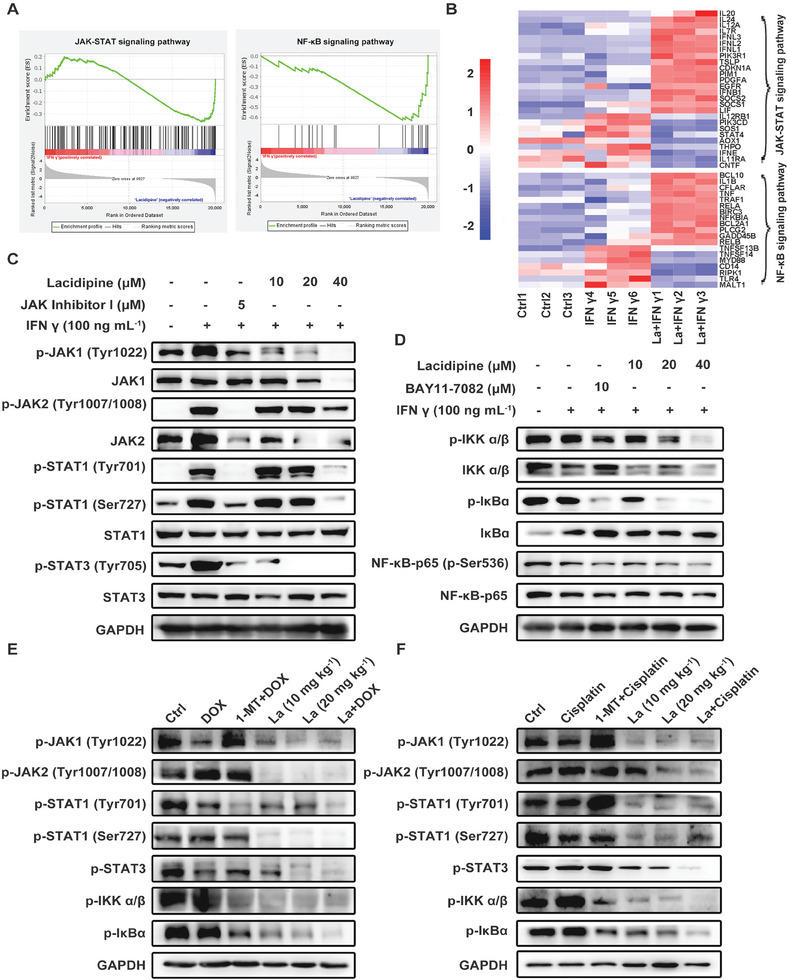
Lacidipine inhibits the IDO1 expression through suppression of JAK/STAT and NF‐κB signaling pathways. A) Gene set enrichment analysis (GSEA) for JAK/STAT and NF‐κB signaling pathways. Rank statistics (bottom; see y axis) and normalized enrichment scores (top; see y axis) indicate downregulation and upregulation, respectively. JAK/STAT signaling pathways, normalized enrichment score (NES) = −1.37, *p* < 0.001, FDR *q* = 0.11; NF‐κB signaling pathways, NES = ‐1.64, *p* = 0.01, FDR *q* = 0.17. B) Heat map hierarchical clustering displays the differentially expressed genes in the JAK/STAT and NF‐κB signaling pathways with a *p* < 0.05 (*n* = 3, each group). La, Lacidipine. C) MDA‐MB‐231 cells were pre‐treated with lacidipine (10 × 10^−6^, 20 × 10^−6^, and 40 × 10^−6^ m) and JAK inhibitor I (5 × 10^−6^ m) for 2 h, then stimulated with IFN γ (100 ng mL^−1^) for 12 h. The expression of key proteins of the JAK/STAT signaling pathway was analyzed by western blotting. GAPDH was used as the loading control. D) MDA‐MB‐231 cells were pre‐treated with lacidipine (10 × 10^−6^, 20 × 10^−6^, and 40 × 10^−6^ m) and BAY11‐7082 (10 × 10^−6^ m) for 2 h, then stimulated with IFN γ (100 ng mL^−1^) for 12 h. The expression of key proteins of the NF‐κB signaling pathway was analyzed by western blotting. GAPDH was used as the loading control. E,F) The the key proteins expression of the JAK/STAT and NF‐κB signaling pathways were analyzed by western blotting in mouse tumor tissue homogenate of at the end of treatment. GAPDH was used as the loading control. All western blot experiments were conducted with three independent replicates.

The transcriptional landscape was analyzed to reveal the underlying mechanism of lacidipine in the regulation of IDO expression. Kyoto Encyclopedia of Genes and Genomes (KEGG) pathway clustering analyses indicated that lacidipine regulated immune‐related signaling pathways, such as the p53 signaling pathway, Th1 and Th2 cell differentiation, and antigen processing and presentation (Figure S5A, Supporting Information). In addition, lacidipine modulated the expression of differentially expressed genes and downregulated the JAK/STAT and NF‐κB signaling pathways (**Figure** **5**A,B). Consistent with this, lacidipine significantly inhibited the JAK/STAT and NF‐κB signaling pathways by suppressing the phosphorylation of JAK1, JAK2, STAT1, STAT3, IKKα/β, IκBα, and NF‐κB‐p65 proteins induced by IFN γ and upregulated the expression of IκBα in MDA‐MB‐231, MCF‐7, and 4T1 cells at 12 h (Figure 5C,D and Figure S5B,C, Supporting Information) and 24 h (Figure S5D–I, Supporting Information). Interestingly, lacidipine alone also inhibited the JAK/STAT and NF‐κB signaling pathways at 24 h (Figure S5J–M, Supporting Information). Next, we examined the expression of relative proteins in the homogenate of mouse 4T1 tumor tissues, and the results showed that treatment with lacidipine alone and in combination with DOX/cisplatin significantly inhibited the phosphorylation of key proteins in JAK/STAT and NF‐κB signaling pathway (Figure 5E,F). Taken together, these results suggest that lacidipine regulates IDO expression by inhibiting the JAK/STAT and NF‐κB signaling pathways.

### 2.6 Ca^2+^ Participates in Lacidipine Regulation of IDO1 Expression

**Figure 6:**
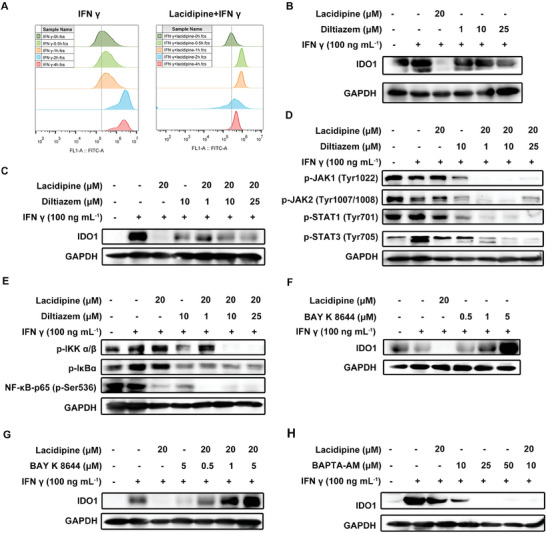
Ca^2+^ participates in lacidipine regulation of IDO1 expression. A) Effect of IFN γ only and lacidipine + IFN γ on intracellular Ca^2+^ concentration. MDA‐MB‐231 cells were pre‐treated with lacidipine (20 × 10^−6^ m) for 2 h, and then stimulated with IFN γ (100 ng mL^−1^) for different durations (0, 0.5, 1, 2, and 4 h). The cells were loaded with 5 × 10^−6^ m fluo‐3 AM, and intracellular Ca^2+^ levels were analyzed using flow cytometry. B–E) MDA‐MB‐231 cells were pre‐treated with lacidipine (20 × 10^−6^ m) and diltiazem (1 × 10^−6^, 10 × 10^−6^, 25 × 10^−6^ m) for 2 h, then stimulated with IFN γ (100 ng mL^−1^) for 24 h. The expression of B,C) IDO1 proteins and D) the key proteins of the JAK/STAT and E) NF‐κB signaling pathways were analyzed by western blotting. GAPDH was used as the loading control. F,G) MDA‐MB‐231 cells were pre‐treated with lacidipine (20 × 10^−6^ m) and BAY K 8644 (0.5 × 10^−6^, 1 × 10^−6^, and 5 × 10^−6^ m) for 2 h, then stimulated with IFN γ (100 ng mL^−1^) for 24 h. The expression of IDO1 proteins were analyzed by western blotting. GAPDH was used as the loading control. H) MDA‐MB‐231 cells were pre‐treated with lacidipine (20 × 10^−6^ m) and BAPTA‐AM (10 × 10^−6^, 25 × 10^−6^, and 50 × 10^−6^ m) for 2 h, then stimulated with IFN γ (100 ng mL^−1^) for 24 h. The expression of IDO1 proteins was analyzed using western blotting. GAPDH was used as the loading control. All experiments were conducted with three independent replicates.

As a Ca_V_1 channel inhibitor, lacidipine modulates intracellular Ca^2+^ concentration. To examine whether the effect of lacidipine on IDO1 was related to intracellular free Ca^2+^ concentration, MDA‐MB‐231 cells were pre‐treated with IFN γ, and lacidipine plus IFN γ to detect intracellular Ca^2+^ concentration changes using the Ca^2+^ probe Fluo‐3 AM. IFN γ increased intracellular Ca^2+^ concentration. In comparison, lacidipine plus IFN γ co‐treatment initially increased intracellular Ca^2+^ concentration, but significantly inhibited intracellular Ca^2+^ concentration at 2 h (**Figure** **6**A). Diltiazem alone significantly downregulated the IFN γ induced IDO1 expression in MDA‐MB‐231 and MCF‐7 cells (Figure 6B and Figure S6A, Supporting Information) and inhibited the activation of the JAK/STAT and NF‐κB signaling pathways (Figure 6D,E). However, diltiazem enhanced the inhibitory effect of lacidipine on the phosphorylation of JAK1/2, STAT1/3, IKKα/β, and NF‐κB‐p65, but had no similar effect on IDO1 and IκBα phosphorylation (Figure 6C–E). It is possible that lacidipine completely closed calcium channels, maintaining intracellular Ca^2^⁺ at a low equilibrium state that cannot be further decreased by diltiazem. To further confirm the role of intracellular Ca^2+^ in the regulation of IDO1 expression, Bay K 8644, an agonist of Ca_V_1 channels, was used to stimulate the intracellular Ca^2+^ concentration. Treatment with Bay K 8644 alone upregulated IFN γ‐induced IDO1 expression (Figure 6F and Figure S6B, Supporting Information). Bay K 8644 also alleviated the inhibitory effect of lacidipine on IDO1 expression (Figure 6G). In addition, the addition of BAPTA‐AM, a calcium chelator, further enhanced the inhibitory effect of lacidipine on IDO1 expression in MDA‐MB‐231 cells (Figure 6H). Taken together, these results suggest that intracellular Ca^2+^ levels mediate the effects of lacidipine on the expression of IDO1.

### 2.7 Ca_V_1.2/1.3 Regulates IDO1 Expression via Pyk2 and Calmodulin

**Figure 7:**
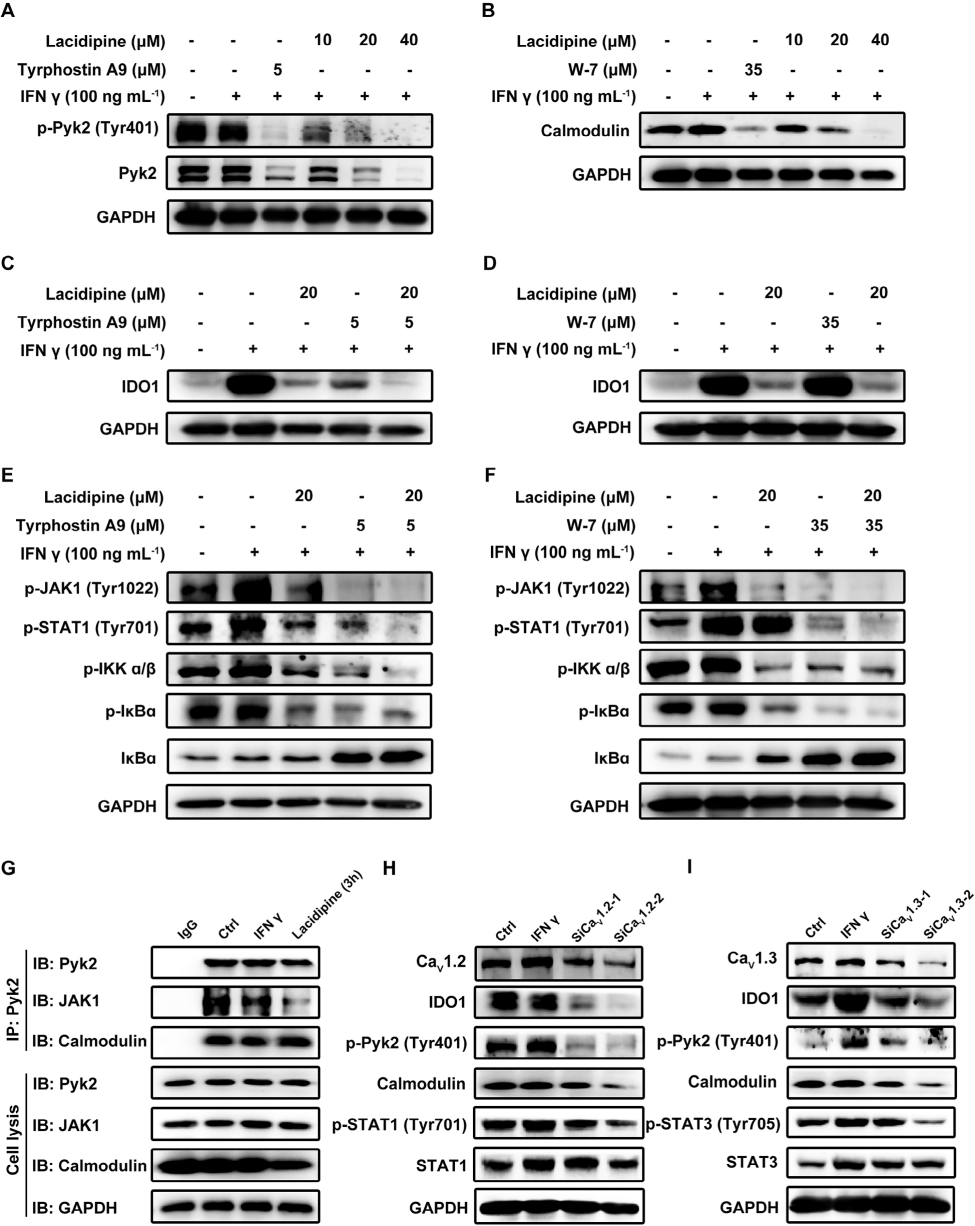
Ca_V_1.2/1.3 regulates IDO1 expression via Pyk2 and calmodulin. A,B) MDA‐MB‐231 cells were pre‐treated with lacidipine (10 × 10^−6^, 20 × 10^−6^, and 40 × 10^−6^ m) and tyrphostin A9 (5 × 10^−6^ m) or W‐7 (35 × 10^−6^ m) for 2 h, then stimulated with IFN γ (100 ng mL^−1^) for 24 h. The expression of Pyk2 phosphorylated at A) Tyr402, Pyk2 and B) calmodulin (B) proteins was analyzed by western blotting. GAPDH was used as the loading control. MDA‐MB‐231 cells were pre‐treated with lacidipine (20 × 10^−6^ m) and C) tyrphostin A9 (5 × 10^−6^ m) or D) W‐7 for 2 h, then stimulated with IFN γ (100 ng mL^−1^) for 24 h. The expression of IDO1 proteins was analyzed by western blotting. GAPDH was used as the loading control. MDA‐MB‐231 cells were pre‐treated with E) lacidipine (20 × 10^−6^ m) and tyrphostin A9 (5 × 10^−6^ m) or F) W‐7 for 2 h, then stimulated with IFN γ (100 ng mL^−1^) for 24 h. The expression of phosphorylated JAK at Tyr1022, phosphorylated STAT1 at Tyr701, phosphorylated IKK α/β, phosphorylated IκBα, and IκBα proteins was analyzed by western blotting. GAPDH was used as the loading control. G) MDA‐MB‐231 cells were treated with lacidipine (20 × 10^−6^ m) for 3 h and then co‐immunoprecipitated with Pyk2 antibodies or control immunoglobulin G (IgG) and analyzed for antibody‐specific JAK1, calmodulin, and Pyk2. The MDA‐MB‐231 cells were transfected with the indicated H) Ca_V_1.2 and I) Ca_V_1.3 siRNA. After transfection for 48 h, cells were treated with IFN γ (100 ng mL^−1^) for 24 h and cell lysates were collected for western blot analysis using the indicated antibodies to assess the effect of Ca_V_1.2 and Ca_V_1.3. GAPDH was used as the loading control. All experiments were conducted with three independent replicates.

Previous studies have shown that Pyk2 interacts with JAK1 to participate in the calcium‐regulated JAK/STAT activation in macrophages.^[^ ^17^ ^]^ Calmodulin, a key Ca^2^⁺ receptor, interacts with Pyk2 to regulate its activation^[^ ^29^ ^]^ and is also involved in the phosphorylation of IκBα, a key step in NF‐κB activation.^[^ ^30^ ^]^ Therefore, we hypothesized that Pyk2 is critical for lacidipine‐regulated intracellular Ca^2+^ homeostasis and IDO1 expression. Indeed, lacidipine markedly suppressed Pyk2 phosphorylation and the expression of calmodulin regardless of the presence of IFN γ in a dose‐ and time‐dependent manner in MDA‐MB‐231 and MCF‐7 cells, similar to tyrphostin A9 and W‐7, inhibitors of Pyk2 and calmodulin, respectively (**Figure** **7**A,B and Figure S6C–F, Supporting Information). Furthermore, lacidipine treatment alone and in combination with DOX/cisplatin inhibited Pyk2 phosphorylation in 4T1 mouse tumors (Figure S6G–J, Supporting Information). Tyrphostin A9 and W‐7 also inhibited IFN γ‐induced IDO1 expression, which further enhanced the inhibitory effect of lacidipine on IDO1 expression (Figure 7C,D and Figure S6K,L, Supporting Information). Tyrphostin A9 and W‐7 not only significantly inhibited IFN γ‐stimulated phosphorylation of JAK1, STAT1, IKKα/β, and IκBα and increased the expression of IκBα, but also potentiated the inhibitory effect of lacidipine on these proteins (Figure 7E,F and Figure S6M,N, Supporting Information). Furthermore, we examined the effect of lacidipine on the intracellular physical association of Pyk2 with JAK1 and calmodulin using co‐immunoprecipitation assays. As shown in Figure 7G and Figure S6O (Supporting Information), lacidipine markedly suppressed the interaction between Pyk2 and JAK1, but had no effect on the interaction between Pyk2 and calmodulin in MDA‐MB‐231 and MCF‐7 cells. These results suggest that lacidipine suppresses the participation of calmodulin and Pyk2 in the regulation of IDO1 expression.

Previous studies have shown that mRNA levels of Ca_V_1 channels subtypes Ca_V_1.2 (*CACNA1C*) and Ca_V_1.3 (*CACNA1D*) are highly expressed in breast cancers.^[^ ^31^ ^]^ Consistently, the protein levels of Ca_V_1.2 and Ca_V_1.3 (Figure S7A–C, Supporting Information) and the mRNA levels of *CACNA1C* and *CACNA1D* (Figure S7D, Supporting Information) were detected in MDA‐MB‐231, MCF‐7, and 4T1 cells. To further elucidate the role of Ca_V_1.2 and Ca_V_1.3, in the regulation of IDO1 expression, we knocked down Ca_V_1.2 and Ca_V_1.3 in MDA‐MB‐231 cells. As shown in Figure 7H,I and Figure S7E (Supporting Information), the knockdown of Ca_V_1.2 and Ca_V_1.3 inhibited the expression of key proteins, including IDO1 and calmodulin, and inhibited the phosphorylation of Pyk2, JAK1/2, IKKα/β, and IκBα, similar to the effects observed with lacidipine. In addition, knockdown of Ca_V_1.2 alone inhibited STAT1 tyrosine phosphorylation, whereas knockdown of Ca_V_1.3 inhibited STAT3 tyrosine phosphorylation, indicating that lacidipine simultaneously interacted with Ca_V_1.2 and Ca_V_1.3 to exert its effect. These results suggest that lacidipine concurrently targets Ca_V_1.2 and Ca_V_1.3 to inhibit the formation of Pyk2/JAK1/calmodulin complex, leading to the inactivation of JAK/STAT and NF‐κB signaling pathway and subsequent IDO1 expression.

### 2.8 The Expression Levels of IDO1 and Pyk2 Phosphorylation Correlate with Human Breast Cancer Development

**Figure 8:**
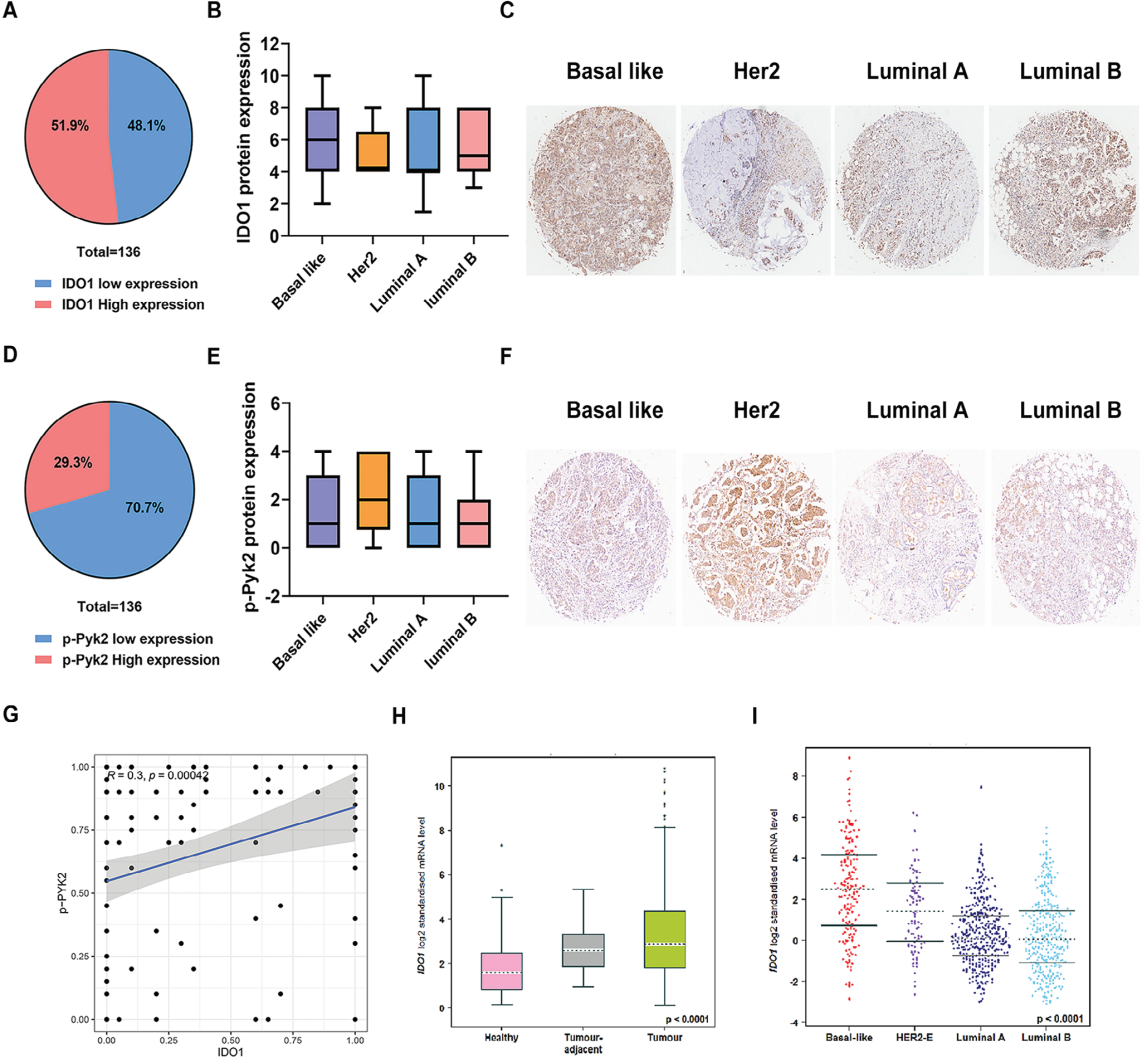
The expression levels of IDO1 and Pyk2 phosphorylation correlate with human breast cancer development. A) Statistical analysis of IDO1 expression in the breast tumor tissues (*n* = 136). B,C) Expression of IDO1 in different subtypes of breast cancer (*n* = 136). D) Statistical analysis of Pyk2 phosphorylation expression in the breast tumor tissues (*n* = 136). E,F) Expression levels of Pyk2 phosphorylation in different subtypes of breast cancer (*n* = 136). G) The correlation of IDO1 and Pyk2 phosphorylation, *R* = 0.3, *p* = 0.00042. H) Expression levels of IDO1 in the breast tumor tissues, adjacent normal breast tissues, and normal breast tissue based on TCGA data (*n* = 1034). I) Expression levels of IDO1 in different subtypes of breast cancer based on TCGA data, *p* < 0.001 (*n* = 1034).

To clarify the clinical significance of IDO1 and Pyk2 phosphorylation in the progression of breast cancer, we examined the protein expression of IDO1 and Pyk2 phosphorylation in the tumor tissues of 136 patients with breast cancer. Immunohistochemical analysis showed that 51.9% of patients exhibited high IDO1 expression, while 48.1% patients showed low IDO1 expression (**Figure** **8**A). Notably, the highest IDO1 levels were observed in the basal‐like subtype, which is predominant in TNBC (Figure 8B,C). In addition, patients with high and low expression of Pyk2 phosphorylation accounted for 29.3% and 70.7% of all patients, respectively (Figure 8D), with the highest the level of Pyk2 phosphorylation in the Her2 subtype (Figure 8E,F). Importantly, IDO1 expression was positively correlated with Pyk2 phosphorylation (*R* = 0.3, *p* = 0.00042) in 136 patients with breast cancer (Figure 8G). To further validate our results, we analyzed breast cancer data based on The Cancer Genome Atlas (TCGA) (*n* = 1034). The mRNA expression of *IDO1* in breast tumor tissues was higher than that in normal and paracancerous tissues (*p* < 0.0001) (Figure 8H). Consistent with our results, the mRNA expression of *IDO1* was highest in the invasive basal‐like subtype of breast cancer (Figure 8I). There were positive correlations between Ca_V_1.2 and IDO1 (*R* = 0.21, *p* = 0.0055), Pyk2 and IDO1 (R = 0.21, *p* <0.0001) (Figure S7F, Supporting Information). Compared with normal and paracancerous tissues, the strong expression of IDO in breast tumor tissues may be an independent diagnostic factor for the efficacy of lacidipine in the treatment of TNBC.

## 3 Discussion

Hypertension and cancer share overlapping biological pathways, as evidenced by the anti‐cancer potential of certain antihypertensive medications. For example, the antihypertensive agents of β‐adrenergic blockers enhance the efficacy of the anthracycline doxorubicin by reducing the development of metastasis, thereby improving cancer survival in patients with TNBC;^[^ ^32^ ^]^ α2‐adrenergic receptor agonist antihypertensive agents exert anti‐tumor immunotherapeutic effects by modulating the function of tumor‐associated macrophages and CD4^+^ T cells.^[^ ^33^ ^]^ Among the commonly prescribed antihypertensive drugs, dihydropyridine calcium channel inhibitors such as amlodipine, nicardipine, and nimodipine have been found to decrease BrdU incorporation into nucleic acids, while amlodipine specifically inhibits cell cycle G1 phase arrest and EGFR tyrosine phosphorylation, leading to reduced proliferation of human epidermal cancer cells.^[^ ^11^, ^34^, ^35^ ^]^ Although antihypertensive drugs such as calcium channel blockers exhibit potential anti‐tumor effects, their role in tumor immunotherapy has been neglected. Owing to the high immunogenicity of TNBC and the significance of immunological surveillance in tumor prevention and development, immunotherapy is becoming an innovative and promising treatment strategy for TNBC. IDO1 is a potent immunomodulatory enzyme that induces a rapid depletion of tryptophan to suppress effector T cell activity and proliferation. On the other hand, it promotes the accumulation of kynurenine, thereby inducing the differentiation of regulatory T cells (Tregs). Differentiation of Tregs inhibits the activation of effector T cells, ultimately leading to local immunosuppression in the tumor microenvironment.^[^ ^36^ ^]^ Therefore, inhibition of IDO presents a promising immunotherapy strategy for breast cancer by alleviating immunosuppression within the tumor microenvironment. Our study demonstrated that the dihydropyridine antihypertensive drug lacidipine acts as a bifunctional modulator of IDO and is involved in IDO‐mediated tumor immunity by inhibiting both the expression and enzymatic activity of IDO in breast cancer. Importantly, IDO1‐mediated immunosuppression is not only associated with its enzyme activity, and IDO1 may also serve as a signaling molecule to maintain or mediate immune tolerance. Therefore, concurrently inhibiting both IDO1 expression and activity may yield a more effective anti‐tumor response than using IDO enzyme activity inhibitors alone, supported by our findings that lacidipine significantly enhanced T cell proliferation.

Chemotherapy effectiveness in TNBC and Her2 breast cancer can be augmented through the involvement of the immune system.^[^ ^37^ ^]^ IDO inhibition has been shown to synergize with various chemotherapeutic agents, improving efficacy in chemotherapy‐refractory breast cancer.^[^ ^38^ ^]^ Our study demonstrated that lacidipine, especially at clinically used doses (0.5 mg kg^−1^ and 1 mg kg^−1^), significantly enhanced the antitumor effects of the chemotherapeutic agents DOX or cisplatin. In addition, selective IDO1 blockade may not be sufficient to alleviate tumor immunosuppression, and IDO2/TDO may serve as an enzyme with a potential compensatory mechanism to attenuate the efficacy of epacadostat, a possible reason for its failure of phase III clinical trial. In this study, we found that lacidipine and other dihydropyridine antihypertensive drugs significantly inhibited the activity and expression of IDO1/IDO/TDO2 both in vitro and in vivo. This suggests that these antihypertensive agents may serve as promising anti‐tumor immunotherapy strategies, potentially outperforming existing IDO1 inhibitors. This is supported by our observation that lacidipine has better anti‐tumor immunomodulatory effects than 1‐MT (BMS‐986205), which has been shown to reduce serum kynurenine levels by 50–60% in combination with nivolumab, an anti‐PD1 antibody.^[^ ^22^ ^]^ Similarly, lacidipine, alone or in combination with chemotherapeutic drugs remarkably inhibited Kyn/Try levels in mouse serum. However, slight refractory tumor growth was observed between days 16 and 25 in the treatment of lacidipine alone and 1‐MT combined with chemotherapeutic agent DOX, indicating partial resistance to the therapy. In contrast, lacidipine, especially at clinically used doses, combined with DOX or cisplatin, demonstrated sustained tumor suppression. Therefore, it should be acknowledged that the inhibition of IDO alone may induce the refractory tumor growth in the treatment of TNBC, which may be obviated by lacidipine in combination with DNA‐inducing agents.^[^ ^39^ ^]^

Another possible reason for the clinical failure of epacadostat is that the blockade of the Kyn pathway regulates metabolic adaptation in the tumor microenvironment and triggers the activation of alternative NAD generation pathways driven by multiple mechanisms, including increasing the mRNA expression of various enzymes in the NAD synthesis pathway, such as *NAPRT*, *NADSYN*, *NMNAT*, *SLC7A5*. This promotes the production of phosphoribosyl pyrophosphate (PRPP), which serves as a co‐substrate for NAD synthesis along all three key NAD biosynthetic pathways.^[^ ^40^ ^]^ Additionally, it promotes the cytoplasmic oxidation of NADH to NAD via compensatory upregulation of the malate‐aspartate shuttle driven by TCA cycle intermediates. Collectively, increased NAD levels in the tumor microenvironment negatively affect the proliferation and function of CD8^+^ T cells.^[^ ^23^ ^]^ In contrast, lacidipine increased the infiltration of CD45^+^CD8^+^ T cells and inhibited CD4^+^CD25^+^Foxp3^+^ Tregs in tumors. Metabolomics showed that lacidipine downregulated the key metabolites in the IFN γ‐mediated Trp metabolic pathway, including N‐formyl‐L‐kynurenine, L‐kynurenine, and 5‐hydroxy‐L‐trypotophan, which was accompanied by the intracellular accumulation of Trp. In addition, lacidipine downregulated the mRNA expression of *QPRT* and *NADSYN* in the *de nov*o NAD synthesis pathway and maintained the mRNA expression of *NAPRT* in the Preiss–Handler pathway to avoid excessive activation of the alternative pathway of NAD generation, although lacidipine increased the expression of *NMNAT* in the salvage pathway of NAD synthesis. However, lacidipine promoted the production of OAA in the TCA cycle, which was not accompanied by an increase in downstream metabolites and, therefore, did not trigger compensatory upregulation of the malate‐aspartate shuttle. In addition, lacidipine inhibited de novo NAD synthesis by downregulating *SLC7A*5 mRNA expression to redistribute the intracellular and extracellular Trp/3H‐Kyn ratios. Furthermore, lacidipine decreased NAD resynthesis by inhibiting gene expression in the glycolysis pathway. Taken together, lacidipine virtually did not change the total NAD pool. Therefore, we did not observe an immunosuppressive effect caused by excessive NAD‐mediated inactivation of CD8^+^ T cells. Previous research has demonstrated that kynurenic acid exerted immunosuppressive effects by targeting G protein‐coupled receptor 35 (GPR35)^[^ ^41^ ^]^ and quinolinic acid activated NMDA receptors in macrophages and engaged the Foxo1/PPARγ signaling pathway, resulting in the formation of an immunosuppressive microenvironment.^[^ ^42^ ^]^ Although our targeted metabolomics analysis did not detect kynurenine and quinolinic acid, this may be attributed to their low concentrations in MDA‐MB‐231 cells. However, our findings indicated that lacidipine downregulated kynurenine monooxygenase (*KMO*) and Kynureninase (*KYNU*), potentially leading to reduced levels of the downstream metabolite quinolinic acid. Furthermore, as kynurenic acid and quinolinic acid are downstream metabolites of kynurenine, lacidipine could decrease their levels in vivo and in other tumor cells by inhibiting kynurenine production. Therefore, alongside its role in inhibiting IDO1‐mediated kynurenine‐induced T cell immunosuppression, lacidipine may exert multifaceted immunomodulatory effects by suppressing kynurenic acid and quinolinic acid‐mediated immunosuppression in T cells and macrophages, which needs to be further investigated. Another factor contributing to the failure of clinical trials of epacadostat is the activation of downstream effector pathways involved in Trp metabolism, such as AHR receptors. Our metabolomic results showed that lacidipine had no effect on the metabolites in the Trp‐indole metabolic pathway, thus lacking ligands that activate AHR to play an immunosuppressive role.

IDO1 expression is generally induced by the pro‐inflammatory cytokines IFN γ, TGF‐β, lipopolysaccharides (LPS), and pathogen‐associated molecular pattern (PAMP), and extensively activates IDO1 transcription through NF‐κB and JAK/STAT signaling pathways.^[^ ^16^ ^]^ In this study, we found that lacidipine potently inhibited both the JAK/STAT and NF‐κB signaling pathways. Interestingly, multiple CCBs drugs have also been found to modulate the JAK/STAT and NF‐κB signaling pathways. Amlodipine inhibited the activity of the NF‐κB signaling pathway in spontaneously hypertensive rats and improved ventricular hypertrophy and diastolic function by interfering with the TNF‐α, IL‐1β, and NF‐κB/ IκB inflammatory factor network.^[^ ^43^ ^]^ In addition, nifedipine (10 mg kg^−1^ per day) attenuated DOX‐induced cardiomyocyte apoptosis by blocking L‐type calcium channels, reducing intracellular Ca^2+^ levels, and inhibiting CaMKII‐NF‐κB pathway activation.^[^ ^44^ ^]^ We also found that isradipine, nifedipine, and nitrendipine similarly inhibited IDO activity, with isradipine additionally reducing the expression of IDO1/IDO2/TDO2 in breast cancer cells. This suggests that CCBs play a similar role in regulating the tumor immune response as IDO modulation. Previous studies have reported that increased cellular Ca^2+^ flux promoted STAT1 serine phosphorylation, which is essential for STAT1‐mediated gene transcriptional activation.^[^ ^45^ ^]^ Ca^2+^ regulated the activation balance of STAT1 and STAT3 through the calcium‐dependent kinases CaMK and Pyk2, thereby modulating the inflammatory nature of the cytokine response to a suitable cellular environment in macrophages.^[^ ^17^ ^]^ In addition, Ca^2+^ was closely related to the regulation of NF‐κB signaling; the activation of NF‐κB by endoplasmic reticulum stress requires Ca^2+^ and reactive oxygen intermediates as messenger.^[^ ^46^ ^]^ However, the effect of Ca^2+^ on the JAK/STAT and NF‐κB signaling pathways in breast cancer remains unclear. Our results revealed that the calcium‐dependent tyrosine kinase Pyk2 was essential for linking the calcium pathway with its downstream JAK/STAT and NF‐κB pathways in breast cancer. An increase in intracellular calcium levels facilitated the interaction of calmodulin with Pyk2 and enhanced Pyk2 autophosphorylation and activation, which was pivotal for recruiting other signaling molecules to the complex and transmitting downstream signals, including Src family kinases.^[^ ^29^ ^]^ In addition, Pyk2 interacts with JAK1 to activate the downstream JAK/STAT signaling pathway in macrophages.^[^ ^17^ ^]^ In this study, we showed that Pyk2 formed a complex with calcium‐dependent calmodulin and IFN γ‐related JAK1 in MDA‐MB‐231 and MCF‐7 cells, whereas lacidipine inhibited the interaction of Pyk2 and JAK1 in the complex by reducing the intracellular concentration of Ca^2+^. Calcium channels are the main modulator of cell precisely regulating intracellular Ca^2+^ concentration. However, the effect and mechanism of action of L‐type calcium channels (Ca_V_1 channels) in the regulation of the JAK/STAT and NF‐κB signaling pathways and IDO are unclear. We found that both calcium channels Ca_V_1.2 and Ca_V_1.3 are expressed in breast cancer cell lines, and the knockdown of Ca_V_1.2 and Ca_V_1.3 demonstrated the same effect as lacidipine on the expression of JAK/STAT, NF‐κB signaling pathway‐associated proteins, Pyk2, calmodulin, and IDO. Notably, our study revealed that Ca_V_ channels and Pyk2 regulate IDO through the JAK/STAT and NF‐κB pathways, identifying a previously unrecognized regulatory mechanism of IDO that plays a role in remodeling the tumor microenvironment. It has been shown that Ca_V_β1 regulates T cell function independent of VGCC channel activity.^[^ ^47^ ^]^ Thus, lacidipine exerts immunomodulatory effects by inhibiting IDO in tumor cells, which activates effector T cells and inhibits regulatory T cells, rather than directly acting on T cells themselves.

IDO expression levels have been associated with survival outcomes in several cancers, including prostate, colorectal, and ovarian cancers. Wei et al.^[^ ^48^ ^]^ collected tumor tissues from 65 patients with breast cancer aged 30–79 years for analysis. In univariate analysis, patients with high IDO expression levels and microvessel density tended to have the shortest survival time, which affected PFS and OS. Notably, high Pyk2 expression in triple‐negative breast cancer (TNBC) was strongly correlated with lymph node metastasis and poor outcomes.^[^ ^49^ ^]^ Here, we showed that the expression of IDO1 and Pyk2 phosphorylation were positively correlated in patients with breast cancer. Furthermore, previous studies have shown that Pyk2 promoted EMT, invasion, metastasis, and recurrence in breast cancer by modulating various signaling pathways, suggesting that Pyk2 is one of the most promising metastatic biomarkers in cancer.^[^ ^50^, ^51^ ^]^ Although several Pyk2 inhibitors have been developed and have achieved strong anti‐tumor effects in multiple tumor xenograft models,^[^ ^52^ ^]^ their role in tumor immunity remains unclear. Thus, given the significant potential of IDO1 and Pyk2 as therapeutic targets for breast cancer, further clinical studies are necessary to validate their potential applications in predicting the potential response of CCB to combination immunotherapy. Overexpression of Ca_V_1.2 or Ca_V_1.3 occurs in many cancers including colorectal, gastric, pancreatic, prostate and breast, and their expression and function can be regulated by estrogen and testosterone, correlating with the high incidence of altered expression of these channels in the female and male reproductive systems.^[^ ^53^ ^]^ Here, we found that IDO1 expression correlates with that of Ca_V_1.2 and Pyk2 in human breast cancer samples, consistent with the observation that Ca_V_1.2 exerts the regulatory effect on IDO1 transcription by the Pyk2/JAK1/calmodulin complex. Several epidemiological studies have reported that antihypertensive drugs significantly reduce^[^ ^54^ ^]^ or are not associated with breast cancer risk.^[^ ^55^ ^]^ However, it remains still unknown whether co‐administration of antihypertensive drugs with specific anticancer therapeutics will benefit with the outcome of breast cancer patients. In this study, we found that lacidipine could enhance the anti‐tumor immune effects of chemotherapeutic agents in preclinical models, proposing the possibility that CCBs especially lacidipine can be repurposed to treat particular cancers for patients with hypertension. Additionally, retrospective analyses of cancer patients treated with various therapeutic agents alongside CCBs will be valuable for identifying more effective combination strategies for cancer immunotherapy and advancing drug development targeting tryptophan metabolism.

## 4 Conclusion

In summary, our findings indicated that the antihypertensive drug lacidipine not only directly inhibited the enzymatic activity of IDO1, but also targeted the calcium channels (Ca_V_1.2/1.3) to disrupt the formation of the calcium‐mediated Pyk2‐JAK1‐calmodulin complex, thereby suppressing the JAK/STAT and the Ca^2+^‐dependent calmodulin‐mediated NF‐κB signaling pathways and the subsequent inhibition of IDO transcription. Consequently, lacidipine reprogrammed tryptophan metabolism, effectively decreasing the kynurenine production by suppressing the expression and activity of IDO1/IDO2/TDO2, and maintaining a stable NAD pool, potentially through upregulating *NMNAD* and *NAMPT* and downregulating *NADSYN*, *SLC7A5* in NAD biosynthesis pathway. Ultimately, this activated CD45^+^CD8^+^ effector T cells and incapacitated CD4^+^CD25^+^Foxp3^+^ Tregs, transforming “cold” into “hot” tumors and enhancing the anti‐tumor effect of chemotherapeutic agents in breast cancer in vivo. This study not only reveals a new function of calcium channels in the mediation of tryptophan metabolism, providing new insights for understanding the cross‐talk between hypertension and cancers, but also provides new therapeutic strategies for the clinical use of CCBs to enhance the immune response to chemotherapeutic drugs.

## 5 Experimental Section

### Cell Culture

Human breast cancer cell lines MDA‐MB‐231 and MCF‐7 were purchased from Procell Life Science & Technology Co. Ltd. (Wuhan, China). Human embryonic kidney HEK293A cells and human cervical cancer HeLa cells were obtained from Cell Resource Center of Shanghai Institute for Biological Sciences, Chinese Academy of Sciences (Shanghai, China). Murine luciferase‐transfected 4T1 (4T1‐Luc) breast cancer cells were obtained from Sheng Da Wei Technology Co., ltd. (Wuhan, China). MDA‐MB‐231 cells were cultured in Leibovitz's L15 medium (#LA9510, Solarbio), HEK293A cells were cultured in Dulbecco's modified Eagle's medium (#SH30243.01, Hyclone), 4T1‐Luc cells were maintained in RPMI‐1640 medium (#SH30809.01, Hyclone), and MCF‐7 cells in minimum Eagle's medium (#C11095500BT, Gibco) containing 1% GlutaMAXTM (#35050‐061, Gibco), 1% MEM non‐essential amino acids, 100× (#11140‐050, Gibco), 1% sodium pyruvate 100 × 10^−3^ m Solution (#11360‐070, Gibco) and 0.01 mg mL^−1^ human recombinant insulin (#I5500, Sigma), each supplemented with 10% (v/v) FBS (#10099‐141, Gibco) and 1% penicillin–streptomycin (#C0222, Beyotime) in a humidified, 5% CO_2_‐containing atmosphere incubator at 37 °C. These cells were authenticated by Genetic Testing Biotechnology (Suzhou, China) using short tandem repeat profiling, and examined for mycoplasma contamination.

### IDO1 Cellular Assay

IDO1 cellular assays were performed using standard methods described previously.^[^ ^56^ ^]^ Briefly, MDA‐MB‐231 and MCF‐7 cells were seeded into 96‐well plates. On the following day, the compounds were added to the cells and incubated for 2 h. Then, human recombinant IFN γ (100 ng mL^−1^), (#Z02915, Genscript) was added to the medium containing L‐tryptophan (250 × 10^−3^ m) (# T0011, Solarbio) to induce the expression of IDO1. Cells were incubated at 37 °C in CO_2_ (5%) for 24 h. Supernatants (140 µL) were harvested and mixed with 10 µL of 6.1 N trichloroacetic acid (#T55312, Acmec). The mixture was incubated at 50 °C for 30 min to convert N‐formyl‐L‐kynurenine produced by IDO1 to kynurenine and further centrifuged at 600 × g for 10 min. Removed the precipitation and supernatant (100 µL) was added to 2% (w/v) p‐dimethylaminobenzaldehyde (#sc‐202888, ChemCruz) in acetic acid (100 µL). Absorbance was measured at 480 nm using a Varioskan Flash Multimode Reader (Thermo Fisher). The mean values were calculated from three independent experiments, and the IC_50_ values were determined by non‐linear regression using GraphPad Prism 8.0 software (GraphPad Software Inc., San Diego, CA, USA).

### TDO2 Cellular Assay

The TDO2 cellular assay was conducted following a previously described method with slight modifications.^[^ ^57^ ^]^ The HEK293A cells were transfected with pCMV3‐TDO2‐plasmid using TransIntro EL Transfection Reagent (#FT201, TransGen Biotech) for 24 h, and the transfected cells were treated with various concentrations of compounds for 6 h. Next, cell culture medium (300 µL) was mixed with trichloroacetic acid (90 µL, 30%, w/v) and incubated at 65 °C for 30 min. After centrifugation at 13000 × g for 10 min, an equal volume of 2% (w/v) p‐dimethylaminobenzaldehyde in acetic acid solution was added to the supernatant (100 µL). Absorbance was measured at 480 nm using a Varioskan Flash Multimode Reader (Thermo Fisher). The mean values were calculated from three independent experiments.

### IDO Enzyme Activity Assay

The recombinant IDO1 activity assay was conducted as previously described method with slight modifications.^[^ ^58^ ^]^ In brief, the IDO activity was measured with the standard reaction mixture (250 L) containing potassium phosphate buffer (50 × 10^−3^ m, pH = 6.5), L‐tryptophan (200 × 10^−6^ m), methylene blue (10 × 10^−6^ m) (#7220‐79‐3, Macklin), ascorbic acid (20 × 10^−3^ m) (#A8100, Solarbio), catalase (100 µg mL^−1^) (#C8070, Solarbio), and recombinant expressed IDO1 enzyme (10 µg mL^−1^) (#11650‐H70E, SinoBiological). The substrate was incubated with the test compounds at 37 °C for 60 min and then trichloroacetic acid (50 µL, 30%, w/v) was added to terminate the reaction. The mixture was incubated at 65 °C for 30 min to hydrolyze N‐formyl‐L‐kynurenine produced by IDO1 to kynurenine. After centrifugation at 25 °C at 1500 × *g* for 10 min, the supernatant (100 µL) was mixed with an equal volume 2% (w/v) p‐dimethylaminobenzaldehyde in acetic acid. Absorbance was measured at 480 nm using a Varioskan Flash Multimode Reader (Thermo Fisher). The mean values were calculated from three independent experiments.

### Cell Proliferation Assay

Cell proliferation was examined using a Cell Counting Kit‐8 (CCK‐8) (#B34304, Bimake). Briefly, MDA‐MB‐231, MCF‐7, and HeLa cells were seeded into 96‐well plates and treated with various concentrations of lacidipine for 24 h. Subsequently, 10% CCK solution was added to each well and incubated for 1–4 h. Finally, the absorbance was measured at 450 nm using Varioskan Flash Multimode Reader (Thermo Fisher). The ratio of viability (%) was calculated as ODsample/ODcontrol × 100%. The mean values were calculated from three independent experiments and IC_50_ values were calculated from nonlinear fit curves using GraphPad Prism 8.0 software.

### Western Blot Analyses

Cells were lysed in ice‐cold radioimmunoprecipitation (RIPA) buffer (#P0013D, Beyotime) consist of 1% protease inhibitor cocktail (v/v) (phenylmethylsulfonyl fluoride) (#T505, Beyotime). Total protein from multiple samples was separated by SDS‐PAGE and subsequently transferred onto nitrocellulose membranes (#HATF00010, Merck Millipore). The membranes were blocked in a 5% nonfat powdered milk for 1.5 h at 37 °C, labeled with specific primary antibodies overnight at 4 °C, subsequently rinsed with Tris‐buffered saline containing 0.1% Tween 20 (TBST) four times every 10 min, and incubated with horseradish peroxidase (HRP) conjugated‐secondary antibody (Cell Signaling Technology) for 1.5 h at 37 °C. The protein bands were detected using an ECL detection system (#4AW011‐100, 4A BioTech) on ImageQuant LAS 500 (GE210 Healthcare Bio Sciences AB, USA).

### Real‐Time Quantitative PCR (qRT‐PCR)

Total RNA was extracted with Trizol reagent (#15596026, Invitrogen) and following reverse transcription by TransScript All‐in‐One First‐Strand cDNA Synthesis SuperMix for qPCR (#AT341, TransGen Biotech) detection kit. Subsequently, cDNA was subjected to qRT‐PCR with TransStart Tip Top Green qPCR SuperMix (#AQ141, TransGen Biotech) in a reaction mixture (20 µL) with the following primers: *IDO1*, forward 5′‐AACAGCGCCTTTAGCAAAGTGTCCCGTTCTTG‐3′, reverse 5′‐AGCGCCTTGCACGTCTAGTTCTGGGATGC‐3′; *CYP1A1*, forward, 5′‐CAAGAGGAGCTAGACACAGTGATT‐3′ and reverse, 5′‐AGCCTTTCAAACTTGTGTCTCTTGT‐3′ and *GAPDH*, forward, 5′‐AGGTGAAGGTCGGAGTCAACG‐3′, reverse, 5′‐CCTGGAAGATGGTGATGGGAT‐3′. The levels of *IDO1* and *CYP1A1* relative expression were normalized to that of *GAPDH* using the 2^−ΔΔCt^ method. The results are representative of three independent experiments.

### Cellular Thermal Shift Assay (CETSA)

MDA‐MB‐231 cells treated with IFN γ (100 ng mL^−1^) for 24 h to induce the expression of IDO1. After cells were lysed by repeatedly freezing and thawing three times in liquid nitrogen and water bath (37 °C) and the cell lysate was centrifuged (2000 × g) for 20 min at 4 °C. Subsequently, cell supernatant was collected and treated with indicated concentrations of lacidipine for 30 min at 37 °C. The samples were divided into aliquots for 50 µL, which were incubated at different temperatures (40–65 °C) to denature samples for 3 min, cooled at 37 °C for 3 min, and centrifuged (2000 × *g*) for 20 min at 4 °C. The supernatant was subsequently analyzed by western blotting to evaluate the expression of IDO1.

### Molecular Docking Model

Molecular docking studies were performed on the IDO1 (PDB:4PK5) crystal structure using the AutoDock 4. The structure of lacidipine was determined using ChemDraw software (PerkinElmer). The docking results were obtained based on the combined free energies. Finally, the lowest energy was observed.

### Targeted Metabolomics Analysis

MDA‐MB‐231 cells were treated with DMSO alone (Ctrl), IFN γ (100 ng mL^−1^, IFN γ) for 24 h, and were pre‐treated with lacidipine (20 × 10^−6^ m) for 2 h, then stimulated with IFN γ (100 ng mL^−1^) for 24 h (La + IFN γ). Metabolites in MDA‐MB‐231 cells were extracted and collected for LC‐MS/MS analysis (Applied Protein Technology, Shanghai, China). Briefly, precooled ammonium acetate solution (500 µL, 0.3%) was added to each sample to wash the cells twice, then 1 mL of precooled methanol/acetonitrile/water (2:2:1, v/v/v) solution was added, and the cells were scraped off with a cell spatula and transferred to a 1.5 mL centrifuge tube. The samples were vortexed for 30 s and then sonicated in an ice bath for 30 min. The sample was incubated at ‐20 °C for 1 h to precipitate protein, and centrifuged at 14 000 × *g*, 4 °C for 20 min. Subsequently, cell supernatant was collected and vacuum dried at 37 °C. For the LC‐MS/MS analysis, the samples were redissolved in 100µL acetonitrile/water solution (1:1, v/v) adequately vortexed for 30 s, and then centrifuged at 14 000 × *g*, 4 °C for 15 min. The supernatant was taken for LC‐MS/MS analysis. Analyses were performed using a UHPLC (1290 Infinity LC, Agilent Technologies, Santa Clara, California, USA) coupled with a QTRAP (AB Sciex 5500, Framingham, CT, USA). Energy metabolites (such as those involved in the TCA cycle, glycolysis/gluconeogenesis, purine metabolism, nicotinate and nicotinamide metabolism, and other intracellular metabolites) and Trp metabolites were determined using LC‐MS/MS. The chromatographic peak area and retention time were extracted by Multiquant software, and quantified according to the peak area of the mass spectrum peak.

### RNA‐seq and Data Analysis

Total RNA was extracted from the cells, and mRNA was purified from total RNA using poly T oligo‐attached magnetic beads. An RNA library was constructed using the AMPure XP system (Beckman Coulter, Beverly, MA, USA), and library preparations were sequenced on an Illumina Novaseq platform. Differential expression analysis of ctrl, IFN γ‐treated, and lacidipine + IFN γ‐treated group was performed using the DESeq2 R package (1.20.0). Genes with a *p* value < 0.05, detected by DESeq2, were considered differentially expressed. Detection of differentially expressed genes in KEGG pathway using the clusterProfiler R package. Gene Set Enrichment Analysis (GSEA) was performed using the GSEA analysis tool (http://www.broadinstitute.org/gsea/index.jsp) for functional gene annotation.

### Calcium Analysis

The intracellular calcium concentration was assayed using Fluo‐3 AM according to the manufacturer's instructions (#S1056, Beyotime). MDA‐MB‐231 cells were treated with specific concentrations of compounds for different durations (0, 0.5, 1, 2, and 4 h), then the cells were added to medium containing fluo‐3 AM (5 × 10^−6^ m) and incubated at 37 °C for 30 min for fluorescent probe loading and washed thoroughly. Approximately 20 000 events were recorded, and the fluorescence intensity was determined for flow cytometric analysis using a BD FACScan flow cytometer.

### Immunoprecipitation

MDA‐MB‐231 and MCF‐7 cells were seeded in 10 cm cell culture dishes for 24 h, and the indicated concentrations of compounds were added for 3 h or 12 h. Then, cells were collected and lysed in modified ice‐lysis buffer (20 × 10^−3^ m Tris‐HCl, pH 7.4, 150 × 10^−3^ m NaCl, 10% glycerol, 1% NP‐40, 1 × 10^−3^ m EDTA, and 0.1 × 10^−3^ m protease inhibitor PMSF) and centrifuged (13000 × *g*) for 10 min at 4 °C. Subsequently, cell lysates were incubated with Pyk2 antibodies (2 µg) or normal rabbit immunoglobulin G (IgG) overnight at 4 °C. The following day, protein A/G agarose beads (40 µL, #P2197, Beyotime) was added to the mixed system for 3 h in 4 °C and washed with lysis buffer. The immunoprecipitates were eluted from the beads by boiling in sample buffer. Interacting proteins were separated by sodium dodecyl sulfate‐polyacrylamide gel electrophoresis and subjected to western blotting.

### Database Analysis

The mRNA expression levels of Ca_V_1.2 (*CACNA1C*), Ca_V_1.3 (*CACNA1D*), *IDO1*, and *Pyk2* in normal and tumor tissues and different subtypes of breast cancer were obtained from TCGA database.

### Clinical Sample Analyses

Human breast tissue microarrays (#HBreD136Su02; Shanghai Outdo Biotechnology, Shanghai, China) were used. Antibodies against IDO1 and Pyk2 phosphorylation were used for immunohistochemical staining. The staining intensity of IDO1 and Pyk2 phosphorylation (no staining = 0; weak staining = 1, moderate staining = 2, strong staining = 3) and staining positivity rate (0% = 0, 1‐25% = 1, 26–50% = 2, 51–75% = 3, 76–100% = 4) were scored. The final protein expression score was assessed by multiplying the staining intensity by the positive staining rate.

### siRNA Transfections

MDA‐MB‐231 cells were transfected in OPTI‐MEM media (200 µL, #31985070, Gibco) containing TransIntro EL Transfection Reagent (7 µL) with siRNA targeting Ca_V_1.2 (siRNA1: sense 5′‐GUGGAAUAUCUCUUUUCUCATT ‐3′, anti‐sense 5′‐UGAGAAAGAGAUAUUCCACTT‐3′ and siRNA2: sense 5′‐CUCAAGAUCCCAAGUUCAUTT‐3′, anti‐sense 5′‐AUGAACUUGGGAUCUUGAGTT‐3′) and Ca_V_1.3 (siRNA1: sense 5′‐CGAUACUGGGUUACUUUGATT ‐3′, anti‐sense 5′‐UCAAAGUAACCCAGUAUCGTT‐3′ and siRNA2: sense 5′‐GAACUCUUCGCCUUUCGAATT‐3′, anti‐sense 5′‐UUCGAAAGGCGAAGAGUUCTT ‐3′) or the control. After 24 h of transfection, IFN γ (100 ng mL^−1^) was added to the cells for another 24 h, and the cells were analyzed by western blotting.

### Evaluation of T Cell Proliferation

Naïve T cells were harvested from BALB/c mouse spleens and purified as previously described.^[^ ^59^ ^]^ T cells were pre‐stained with 3 × 10^−6^ m carboxyfluorescein diacetate succinimidyl ester (CFSE) (#S8269, Selleck) at 37 °C for 15 min and then washed three times with PBS. The MDA‐MB‐231, MCF‐7, and 4T1 cells were stimulated with IFN γ (100 ng mL^−1^) and co‐cultured with the CFSE‐labeled T cells at a ratio of 1:5 (cancer cells: T cells). The co‐cultured cells were incubated with anti‐CD3 antibody (100 ng mL^−1^, #100339, BioLegend) and mouse recombinant interleukin (IL)‐2 (10 ng mL^−1^, #212‐12, Peprotech). After 3 d of co‐cultured, T cell proliferation was detected by FACS analysis.

### In Vivo Tumor Experiments

All animal experiments were conducted in accordance with the Good Experimental Practices guidelines adopted by the Chengdu Institute of Biology, Chinese Academy of Sciences. All experimental procedures and animal sample collections were approved by the Committee for Animal Experiments of the Chengdu Institute of Biology, Chinese Academy of Sciences, China (approval number: CIBDWLL2024040).

Female BALB/c mice (6–8 weeks) were injected with 4T1‐Luc cells (1 × 10^5^) by orthotopic transplantation into the mammary fat pads. In vivo imaging was performed 7, 16, and 28 d after orthotopic implantation. Prior to in vivo imaging, the mice were anesthetized with chloroflurane using a small animal anesthetic machine and intraperitoneally injected with a D‐luciferin solution (150 mg kg^−1^) (#ST196, Beyotime). Simultaneous quantification of fluorescence signals using Kodak In‐vivo FX Pro. After the first in vivo imaging, the mice were randomly divided into nine groups (*n* = 12 per group), which were administered the following:1) vehicle, 2) DOX (i. p., 1 mg kg^−1^), 3) 1‐MT (p. o., 1 mg kg^−1^) + DOX (i. p., 1 mg kg^−1^), 4) lacidipine (p. o., 10 mg kg^−1^), 5) lacidipine (p. o., 20 mg kg^−1^), 6) lacidipine (p. o., 20 mg kg^−1^) + DOX (i. p., 1 mg kg^−1^), 7) cisplatin (i. p, 1 mg kg^−1^), 8) 1‐MT (p. o., 1 mg kg^−1^) + cisplatin (i. p., 1 mg kg^−1^), 9) lacidipine (p. o., 20 mg kg^−1^) + cisplatin (i. p., 1 mg kg^−1^). DOX and cisplatin were administered every 3 d, and 1‐MT and lacidipine were administered every 2 d. Tumor volume was measured using a Vernier caliper every 3 d for up to 21 d. The tumor volume was calculated using the following formula: 0.5236 × L1 × (L2)^2^, where L1 and L2 are the long and short tumor axes, respectively. The body weights of the mice were monitored every 3 d. At the end of the experiment, the mice were sacrificed, and blood, tumor tissue, and other experimental organs were collected for further study.

### HPLC Analysis

Methanol (100 µL) was added to mouse serum (100 µL) to precipitate proteins, the precipitate was removed by centrifugation. The supernatant (20 µL) was subjected to analyze by high‐performance liquid chromatography (HPLC)‐diode array detector (DAD) that was equipped with a Agilent Eclipse XDB‐C18 analytic column (250 mm × 4.6 mm, 5 µm). The mobile phase consisted of solvent A (CH_3_CN) and solvent B (H_2_O containing phosphoric acid, pH = 2.3) followed a gradient elution program (0 min, 5% A; 5 min, 15% A; 15 min, 30% A; 20 min, 50% A; 25 min, 80% A; 28 min, 95% A; and 32 min, 95% A) at a flow rate of 1.0 mL min^−1^ at room temperature, monitored by a DAD at 235 nm for L‐kynurenine and 280 nm for L‐tryptophan, respectively.

### Flow Cytometric Analyses

Spleen and tumor tissues were collected for flow cytometric analysis. The tumor tissue was sectioned, then digested with 1 × HBSS buffer containing collagenase I (1 mg mL^−1^, #1904GR001, Biofroxx), collagenase IV (1 mg mL^−1^, #2091, Biofroxx), and DNaseI (0.5 mg mL^−1^, #DN25, Sigma) for 45 min at 37 °C. The cells were filtered through a 70 × 10^−6^ m cell filter and red blood cells were removed using a red blood cell lysate. Purified tumor cells and spleen cells were stained with the APC anti‐mouse CD4 (#100515, Biolegend), PE anti‐mouse CD25 (#102007, Biolegend), PE anti‐mouse CD45 (#103105, Biolegend), and CD8 (#100712, Biolegend) at 37 °C for 30 min. For the staining of intracellular Foxp3, the True‐Nuclear Transcription Factor Buffer Set (#424401, Biolegend) was used for fixation and permeabilization according to manufacturer's instructions, followed by staining with Alexa Fluor 488 anti‐mouse Foxp3 antibody (#126405, Biolegend) at 37 °C for 1 h, the CD45^+^CD8^+^T cells and CD4^+^CD25^+^Foxp3^+^ Tregs were detected using FACS analysis.

### Cytokines Determination by ELISA

ELISA was used to quantify the cytokine levels of transforming growth factor TGF‐β, IFN γ and IL‐10 in mouse serum by using a Mouse ELISA kit (Dakewe) according to manufacturer's instructions. At least three independent experiments were conducted.

### IHC Staining

Tumor tissues were fixed in 4% formaldehyde solution and embedded in paraffin. The embedded tissues were cut into 4 × 10^−6^ m slices and incubated with the antibodies against IDO1 (#13268‐1‐AP, ProteinTech), Pyk2 (Phospho‐Tyr402) (#11216, Signalway), CD8 (#47992, Signalway), and Foxp3 (#AF6544, Affinity) overnight at 4 °C. The tissue sections were then incubated with secondary goat anti‐mouse immunoglobulin G (IgG) and conjugated with a horseradish peroxidase complex. 3,3′‐diaminobenzidine (DAB) was used as a chromogenic agent and the sections were counterstained with hematoxylin. The positively stained cells were observed under a confocal microscope.

### Statistical Analysis

All statistical analyses were performed using Prism 8 (GraphPad Software, La Jolla, CA, USA) and Origin Pro 8 software. The curves were plotted using a variable slope (four‐parameter) non‐linear fit. Statistical significance among experimental groups was evaluated using unpaired two‐tailed Student's *t*‐test and one‐way analysis of variance (ANOVA). *p* values < 0.05 were considered to be statistically significant. All data represent biological replicates (*n*) and were expressed as mean ± SEM.

### Ethics Statement

The animal experiments were conducted in accordance with the guidelines adopted by the Chengdu Institute of Biology, Chinese Academy of Sciences. All experimental procedures and animal sample collections were approved by the Committee for Animal Experiments of the Chengdu Institute of Biology, Chinese Academy of Sciences, China.

## Conflict of Interest

The authors declare no conflict of interest.

## Author Contributions

Y.W.S., X.K.S., L.H.Y., R.Y.X., and J.L.Y. performed the experiments. Y.W.S. and C.Q. conducted the HPLC experiments. Z.H.Z. performed molecular docking. W.L.L. provided reagents and revised the article. F.W. and Y.W.S. analyzed the data and wrote this paper. F.W. and G.L.Z. designed the overall experiments, supervised the research. F.W. and Y.W.S. provided funding.

## Supporting information



Supporting Information

## Data Availability

The data that support the findings of this study are openly available in [the National Genomics Data Center], reference numbers [OMIX004970, HRA005622].
