# Supplementary material for: Calcium Channel Blocker Lacidipine Promotes Antitumor Immunity by Reprogramming Tryptophan Metabolism
Source: Adv Sci (Weinh). 2024 Nov 25;12(3):2409310. doi: 10.1002/advs.202409310 (PMC11744582; doi:10.1002/advs.202409310)
Supplement: Supplementary file 1 — Supporting Information [file ADVS-12-2409310-s001.pdf]

ADVANCED SCIENCE
Open Access

Supporting Information

for *Adv. Sci.*, DOI 10.1002/advs.202409310

Calcium Channel Blocker Lacidipine Promotes Antitumor Immunity by Reprogramming Tryptophan Metabolism

*Yuwen Sheng, Chong Qiao, Zhonghui Zhang, Xiaoke Shi, Linhan Yang, Ruiying Xi, Jialing Yu, Wanli Liu, Guolin Zhang and Fei Wang**

Supporting Information

Calcium Channel Blocker Lacidipine Promotes Anti-tumor Immunity by Reprogramming Tryptophan Metabolism

*Yuwen Sheng, Chong Qiao, Zhonghui Zhang, Xiaoke Shi, Linhan Yang, Ruiying Xi, Jialing Yu, Wanli Liu, Guolin Zhang, Fei Wang**

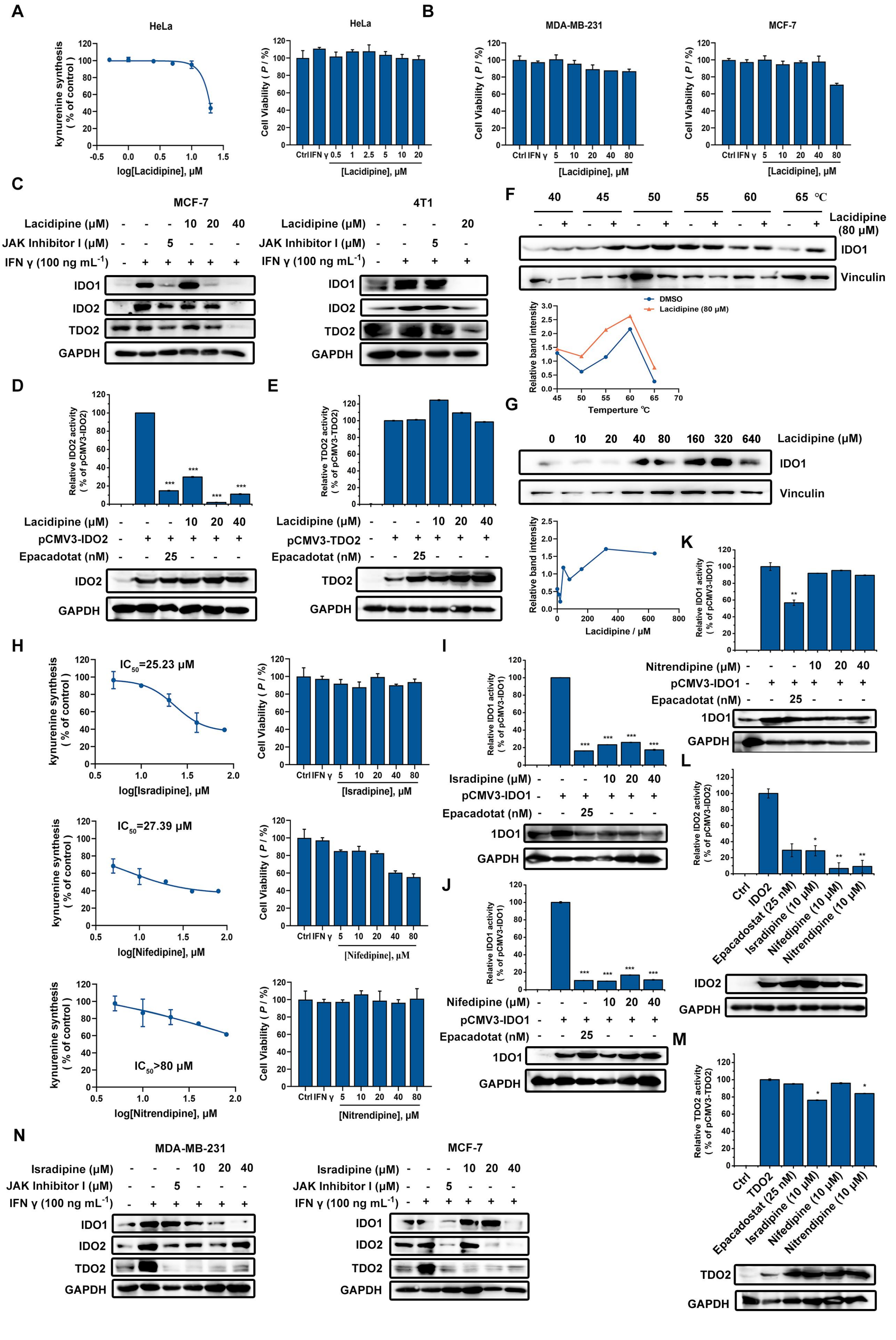
A
B
HeLa
MDA-MB-231
MCF-7
kynurenine synthesis (% of control)
Cell Viability (P / %)
log[Lacidipine], μM
[Lacidipine], μM
C
4T1
Lacidipine (μM)
JAK Inhibitor I (μM)
IFN γ (100 ng mL-1)
IDO1
IDO2
TDO2
GAPDH
D
E
Relative IDO2 activity (% of pCMV3-IDO2)
Relative TDO2 activity (% of pCMV3-TDO2)
pCMV3-IDO2
pCMV3-TDO2
Epacadotat (nM)
F
Lacidipine (80 μM)
Vinculin
DMSO
Relative band intensity
Temperture ℃
G
Lacidipine / μM
H
IC50=25.23 μM
IC50=27.39 μM
IC50>80 μM
log[Isradipine], μM
log[Nifedipine], μM
log[Nitrendipine], μM
[Isradipine], μM
[Nifedipine], μM
[Nitrendipine], μM
I
J
K
Relative IDO1 activity (% of pCMV3-IDO1)
Isradipine (μM)
Nifedipine (μM)
Nitrendipine (μM)
pCMV3-IDO1
L
M
Ctrl
Epacadostat (25 nM)
Isradipine (10 μM)
Nifedipine (10 μM)
Nitrendipine (10 μM)
N

Figure S1. (A) The HeLa cells were treated with lacidipine (0.5, 1, 2.5, 5, 10, and 20 μM) for 2 h, and then treated with IFN γ (100 ng mL^{-1}) for 24 h. The concentration of L-kynurenine in the cell supernatants was determined and cell viability was assessed using the CCK-8 assay. Bars, ± SEM. The curves were plotted using a variable slope (four-parameter) non-linear fit. (B) MDA-MB-231 and MCF-7 cells were treated with indicated concentrations of lacidipine (5–80 μM) for 2 h, and then treated with IFN γ (100 ng mL^{-1}) for 24 h; cell viability was assessed using the CCK-8 assay. Bars, ± SEM. (C) Effect of lacidipine on IDO1 expression. MCF-7 and 4T1 cells were pre-treated with lacidipine (10, 20, and 40 μM) and JAK inhibitor I (5 μM) for 2 h, then stimulated with IFN γ (100 ng mL^{-1}) for 24 h. The expression of IDO1, IDO2, and TDO2 was analyzed by western blotting. GAPDH was used as the loading control. (D, E) HEK293A cells were transfected with pCMV3-IDO2 (D) and pCMV3-TDO2 (E) plasmids for 48 h, and then treated with lacidipine (10, 20, 40 μM) and epacadostat (25 nM) for 6 h. The activities of IDO2 and TDO2 were detected in cell supernatants. Bars, ± SEM. ***p < 0.001 vs. the pCMV3-IDO2 (unpaired two-tailed Student's t-test). Cell lysates were immunoblotted with IDO2 and TDO2 antibodies. GAPDH was used as the loading control. (F, G) CETSA was performed to measure the binding capacity of lacidipine with IDO1. HeLa cells were treated with IFN γ (100 ng mL^{-1}) to induce IDO1 expression for 24 h and treated with lacidipine (80 μM) at various temperatures (F) or added various concentrations of lacidipine at 60°C (G). The stabilizing effect of lacidipine on IDO1 and vinculin was evaluated using western blotting. (H) MDA-MB-231 cells were treated with isradipine, nifedipine and nitrendipine as indicated for 2 h, and then treated with IFN γ (100 ng mL^{-1}) for 24 h. The concentration of L-kynurenine in the cell supernatants was determined and cell viability was assessed using the CCK-8 assay. Bars, ± SEM. The curves were plotted using a variable slope (four-parameter) non-linear fit. (I, J, K) HEK293A cells are transfected with pCMV3-IDO1 plasmids for 48 h, and then treated with isradipine (I), nifedipine (J) and nitrendipine (K) (10, 20, and 40 μM) and epacadostat (25 nM) for 6 h. The activity of IDO1 was detected in cell supernatants. Bars, ± SEM. **p < 0.01 and ***p < 0.001 vs. the pCMV3-IDO1 group (unpaired two-tailed Student's t-test). The cell lysates were immunoblotted with IDO1 antibodies. GAPDH was used as the loading control. (L, M) HEK293A cells are transfected with pCMV3-IDO2 (L) and pCMV3-TDO2 (M) plasmids for 48 h, and then treated with isradipine (10 μM), nifedipine (10 μM), nitrendipine (10 μM) and epacadostat (25 nM) for 6 h. The activities of IDO2 and TDO2 were detected in cell supernatants. Bars, ± SEM. *p < 0.05 and **p < 0.01 vs. pCMV3-IDO2 and pCMV3-TDO2 group (unpaired two-tailed Student's t-test). The cell lysates were immunoblotted with IDO2 and TDO2 antibodies.

GAPDH was used as the loading control. (N) MDA-MB-231and MCF-7 cells were pre-treated with isradipine (10, 20, and 40 μM) and JAK inhibitor I (5 μM) for 2 h, then stimulated with IFN γ (100 ng mL^{-1}) for 24 h. The expression of IDO1, IDO2, and TDO2 was analyzed by western blotting. GAPDH was used as the loading control. All experiments were conducted with three independent replicates.

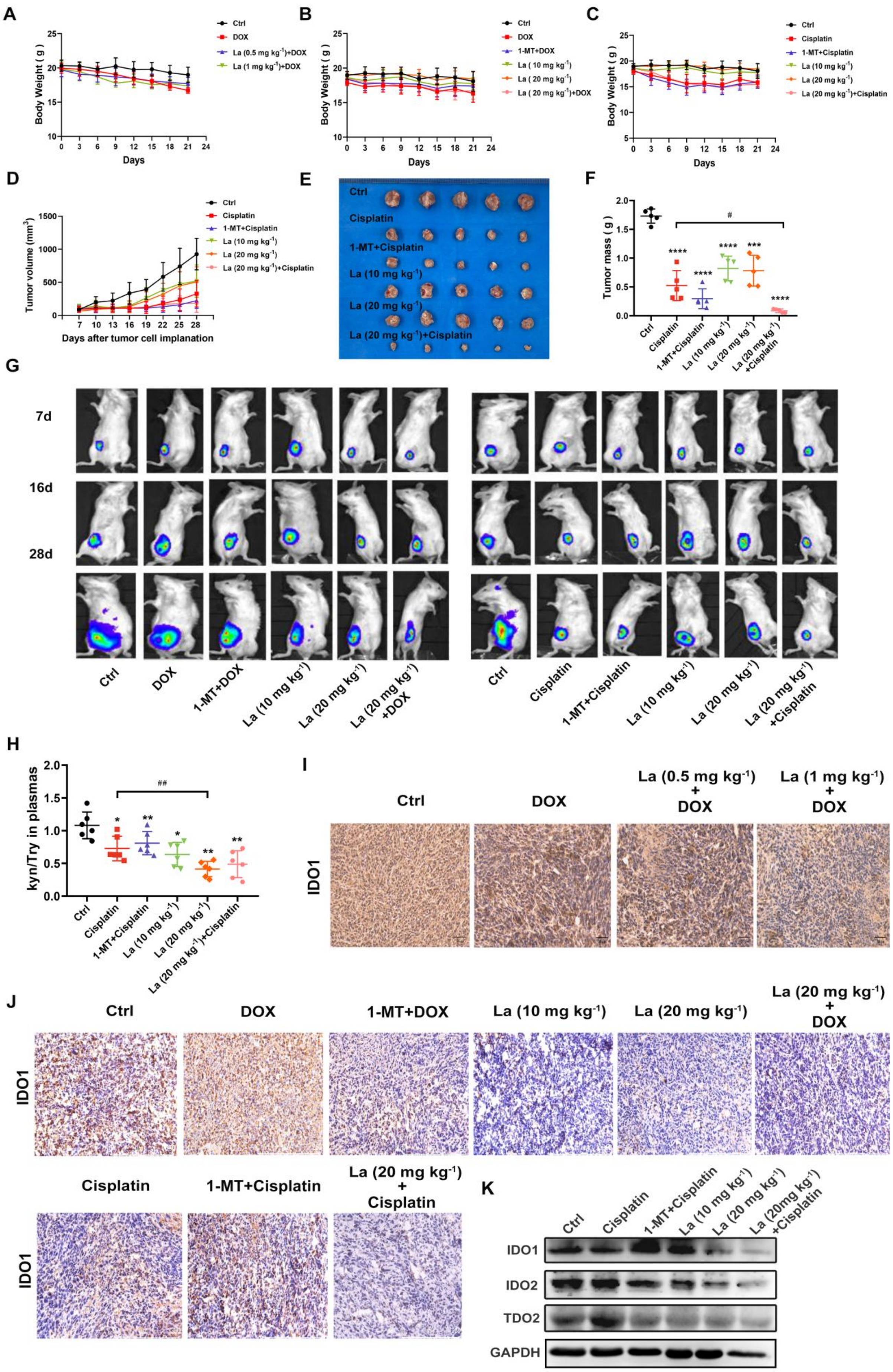
A
Body Weight (g)
Days
Ctrl
DOX
La (0.5 mg kg-1)+DOX
La (1 mg kg-1)+DOX
B
Body Weight (g)
Days
Ctrl
DOX
1-MT+DOX
La (10 mg kg-1)
La (20 mg kg-1)
La (20 mg kg-1)+DOX
C
Body Weight (g)
Days
Ctrl
Cisplatin
1-MT+Cisplatin
La (10 mg kg-1)
La (20 mg kg-1)
La (20 mg kg-1)+Cisplatin
D
Tumor volume (mm3)
Days after tumor cell implanation
Ctrl
Cisplatin
1-MT+Cisplatin
La (10 mg kg-1)
La (20 mg kg-1)
La (20 mg kg-1)+Cisplatin
E
Ctrl
Cisplatin
1-MT+Cisplatin
La (10 mg kg-1)
La (20 mg kg-1)
La (20 mg kg-1)+Cisplatin
F
Tumor mass (g)
Ctrl
Cisplatin
1-MT+Cisplatin
La (10 mg kg-1)
La (20 mg kg-1)
La (20 mg kg-1) +Cisplatin
G
7d
16d
28d
Ctrl
DOX
1-MT+DOX
La (10 mg kg-1)
La (20 mg kg-1)
La (20 mg kg-1) +DOX
Ctrl
Cisplatin
1-MT+Cisplatin
La (10 mg kg-1)
La (20 mg kg-1)
La (20 mg kg-1) +Cisplatin
H
kyn/Try in plasmas
Ctrl
Cisplatin
1-MT+Cisplatin
La (10 mg kg-1)
La (20 mg kg-1)
La (20 mg kg-1)+Cisplatin
I
Ctrl
DOX
La (0.5 mg kg-1) + DOX
La (1 mg kg-1) + DOX
IDO1
J
Ctrl
DOX
1-MT+DOX
La (10 mg kg-1)
La (20 mg kg-1)
La (20 mg kg-1) + DOX
IDO1
Cisplatin
1-MT+Cisplatin
La (20 mg kg-1) + Cisplatin
IDO1
K
Ctrl
Cisplatin
1-MT+Cisplatin
La (10 mg kg-1)
La (20 mg kg-1)
La (20mg kg-1) +Cisplatin
IDO1
IDO2
TDO2
GAPDH

Figure S2. (A-C) Body weight of mice in each treatment group (A: $n = 6$, each group; B-C: $n = 12$, each group). (D) Tumor volumes were recorded in the cisplatin only, lacidipine (10 or 20 mg kg^{-1}) only, and cisplatin + lacidipine (20 mg kg^{-1}) /1-MT groups ($n = 12$, each group) and are presented as the mean ± SEM. (E) Image of the tumors in the cisplatin only, lacidipine (10 or 20 mg kg^{-1}) only, and cisplatin + lacidipine (20 mg kg^{-1}) /1-MT groups ($n = 6$, each group). (F) Tumor mass was recorded in the cisplatin only, lacidipine (10 or 20 mg kg^{-1}) only, and cisplatin + lacidipine (20 mg kg^{-1}) /1-MT groups ($n = 6$, each group) and the results are presented as the mean ± SEM; ***$p < 0.001$ and ****$p < 0.0001$ vs. control group and #$p < 0.05$ vs. cisplatin-treated group (unpaired two-tailed Student's t-test). (G) Tumor growth in the mice was monitored by live imaging to detect the luminescence intensity in mice bearing 4T1-Luc cells on days 7, 16, and 28 ($n = 12$, each group). Representative images are shown. La, Lacidipine. (H) The ratio of Kyn/Try in mouse serum was determined by HPLC at the end of the treatment ($n = 6$, each group). *$p < 0.05$, **$p < 0.01$ vs. control group and ##$p < 0.01$ vs. cisplatin-treated group (unpaired two-tailed Student's t-test). (I, J) The expression of IDO1 was examined by immunohistochemical analysis in tumor tissues ($n = 6$, each group). Representative images are shown. Scale bar = 250 μm. (K) The expression of IDO1, IDO2 and TDO2 were analyzed by western blotting in mouse tumor tissue homogenate of at the end of treatment. GAPDH was used as the loading control. The experiments were conducted with three independent replicates.

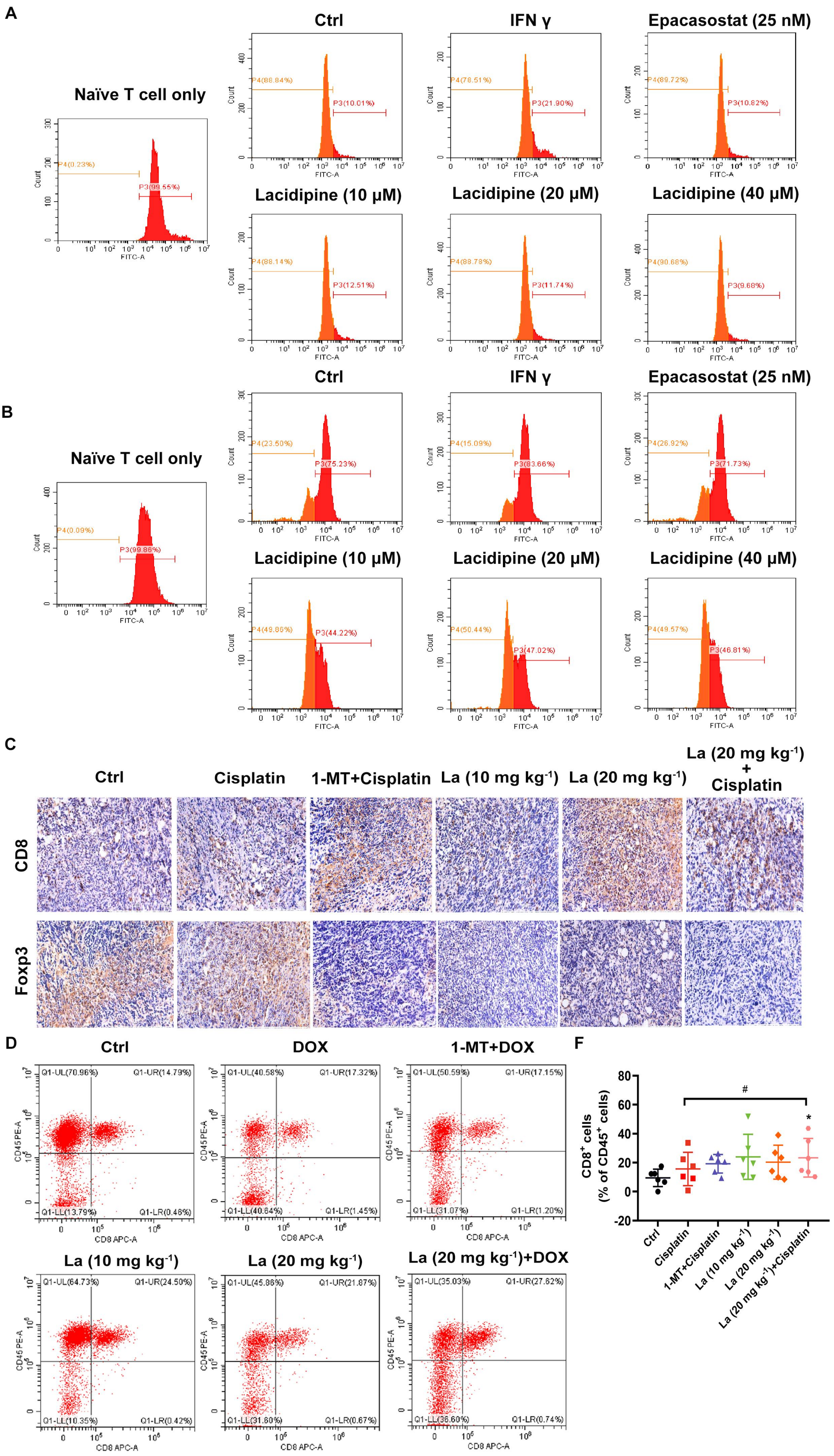
A
Naïve T cell only
Ctrl
IFN γ
Epacasostat (25 nM)
Lacidipine (10 μM)
Lacidipine (20 μM)
Lacidipine (40 μM)
B
Naïve T cell only
Ctrl
IFN γ
Epacasostat (25 nM)
Lacidipine (10 μM)
Lacidipine (20 μM)
Lacidipine (40 μM)
C
Ctrl
Cisplatin
1-MT+Cisplatin
La (10 mg kg-1)
La (20 mg kg-1)
La (20 mg kg-1) + Cisplatin
CD8
Foxp3
D
Ctrl
DOX
1-MT+DOX
La (10 mg kg-1)
La (20 mg kg-1)
La (20 mg kg-1)+DOX
F
CD8+ cells (% of CD45+ cells)
Ctrl
Cisplatin
1-MT+Cisplatin
La (10 mg kg-1)
La (20 mg kg-1)
La (20 mg kg-1)+Cisplatin

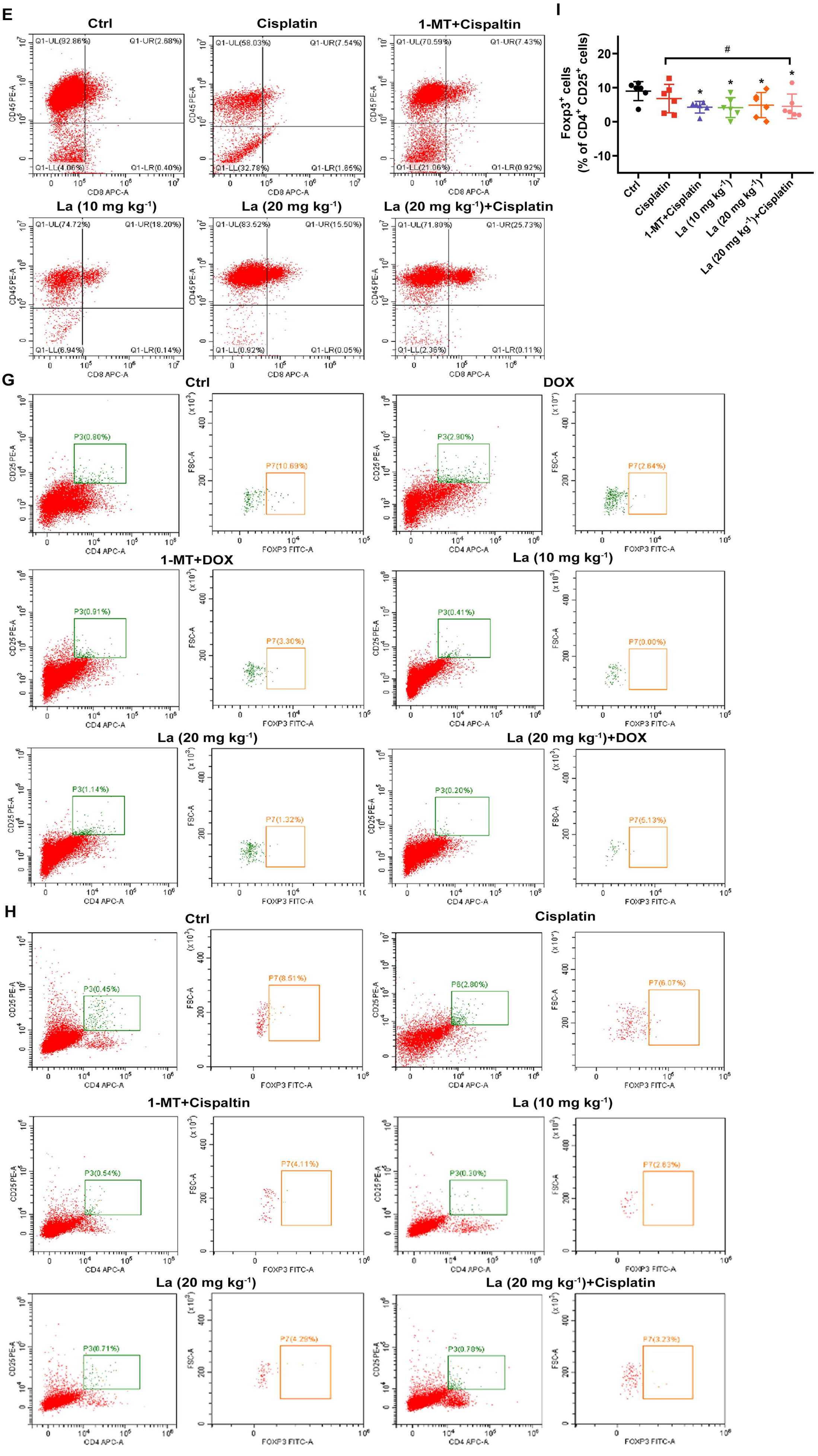
E
Ctrl
Cisplatin
1-MT+Cispaltin
La (10 mg kg-1)
La (20 mg kg-1)
La (20 mg kg-1)+Cisplatin
CD8 APC-A
CD45 PE-A
I
Foxp3+ cells (% of CD4+ CD25+ cells)
Ctrl
Cisplatin
1-MT+Cisplatin
La (10 mg kg-1)
La (20 mg kg-1)
La (20 mg kg-1)+Cisplatin
G
Ctrl
DOX
1-MT+DOX
La (10 mg kg-1)
La (20 mg kg-1)
La (20 mg kg-1)+DOX
CD4 APC-A
CD25 PE-A
FOXP3 FITC-A
FSC-A
H
Ctrl
Cisplatin
1-MT+Cispaltin
La (10 mg kg-1)
La (20 mg kg-1)
La (20 mg kg-1)+Cisplatin

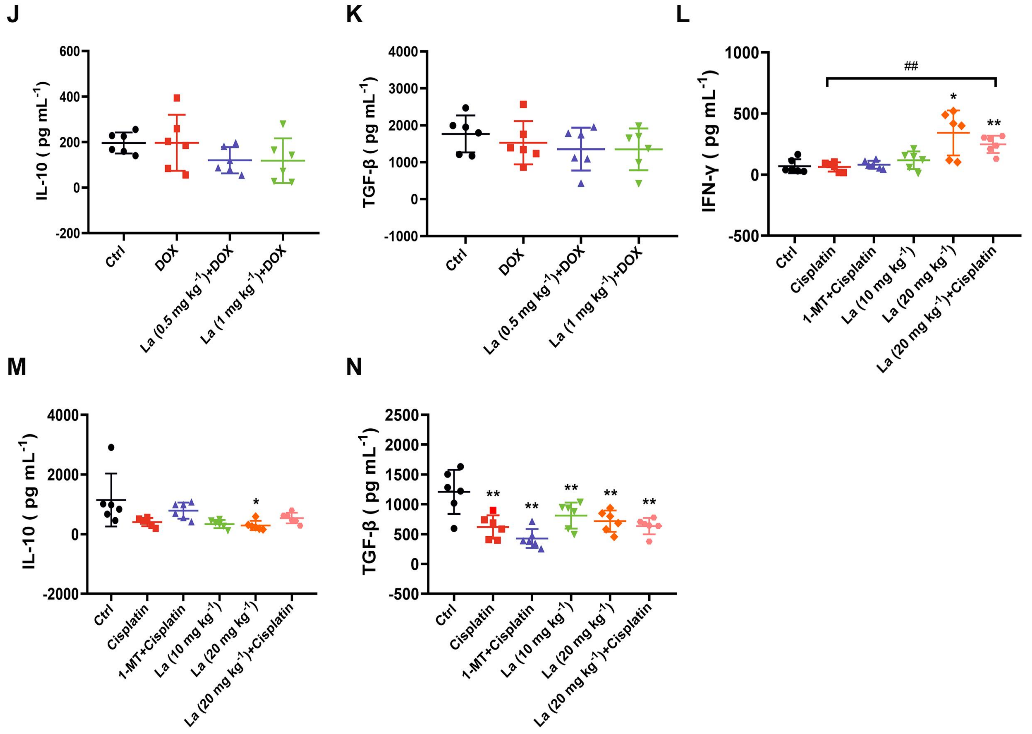

Figure S3. (A, B) Effect of lacidipine on the proliferation of T cells co-cultured with IFN γ-treated MCF-7 (A) and 4T1 cells (B). The proliferation of $CD8^+$ T cells was detected based on CFSE using flow cytometry (FACS). The percentage of proliferating cells is shown. The experiments were conducted with three independent replicates. (C) The expression of CD8 and Foxp3 were examined by immunohistochemical analysis in tumor tissues (n = 6, each group). Representative images are shown. Scale bar = 250 µm. (D-F) Percentage of $CD8^+$ effector T cells in $CD45^+$TILs in tumors was analyzed using flow cytometry at the end of treatment (n = 6, each group) and the results are presented as the mean ± SEM; *p < 0.05 vs. control group and #p < 0.05 vs. cisplatin-treated group (unpaired two-tailed Student's t-test). (G-I) Percentage of $CD4^+CD25^+Foxp3^+$ regulatory T cells in tumors was analyzed using flow cytometry at the end of treatment (n = 6, each group) and the results are presented as the mean ± SEM; *p < 0.05 vs. control group and #p < 0.05 vs. cisplatin-treated group (unpaired two-tailed Student's t-test). (J-N) The levels of cytokines IFN γ, IL-10, and TGF-β were evaluated in tumor tissue homogenates by ELISA (n = 6, each group). *p < 0.05 and **p < 0.01 vs. control group and ##p < 0.01 vs. cisplatin-treated group (unpaired two-tailed Student's t-test). La, Lacidipine.

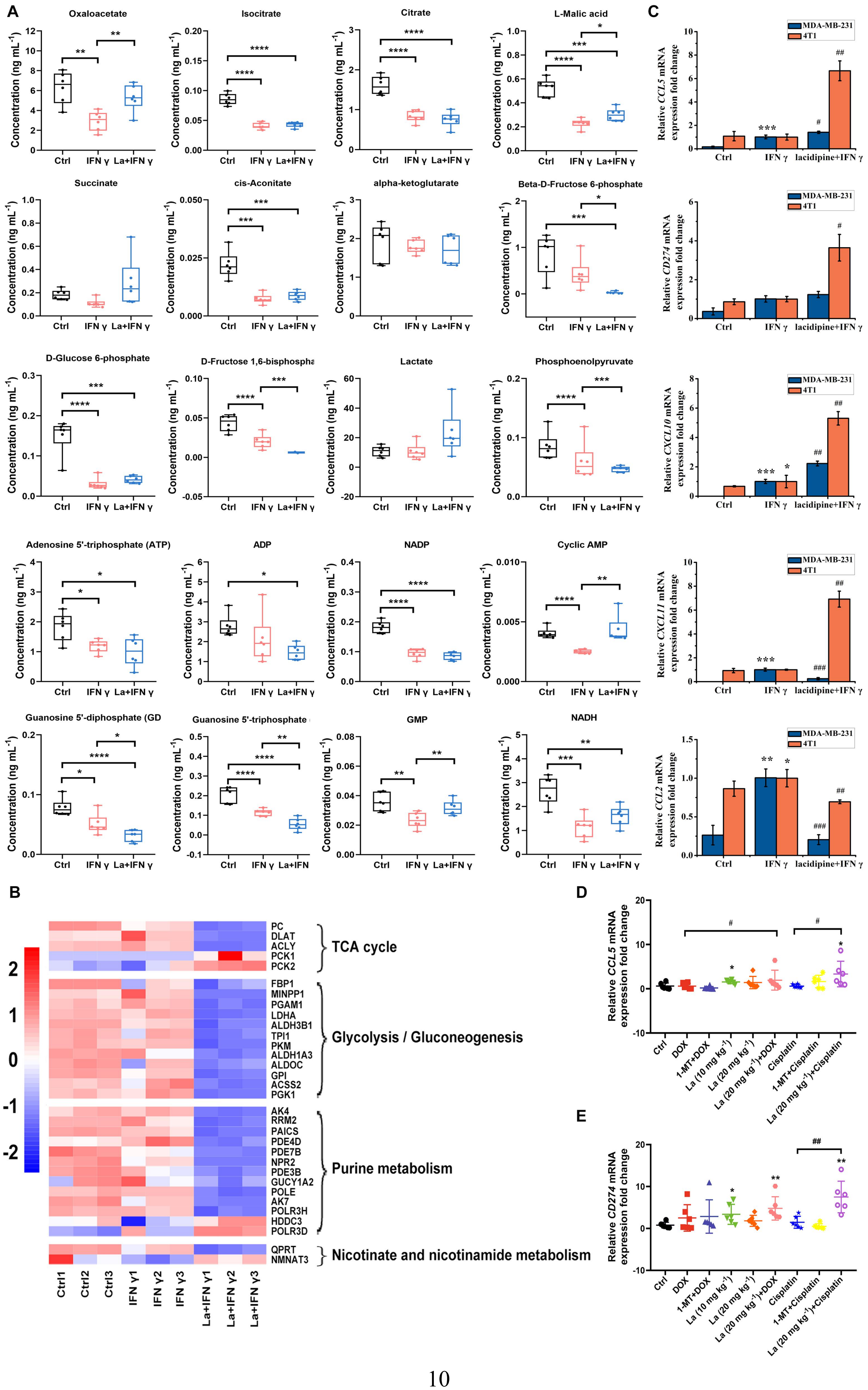
A
Oxaloacetate
Isocitrate
Citrate
L-Malic acid
Succinate
cis-Aconitate
alpha-ketoglutarate
Beta-D-Fructose 6-phosphate
D-Glucose 6-phosphate
D-Fructose 1,6-bisphospha
Lactate
Phosphoenolpyruvate
Adenosine 5'-triphosphate (ATP)
ADP
NADP
Cyclic AMP
Guanosine 5'-diphosphate (GD
Guanosine 5'-triphosphate
GMP
NADH
Concentration (ng mL-1)
Ctrl
IFN γ
La+IFN γ
B
PC
DLAT
ACLY
PCK1
PCK2
TCA cycle
FBP1
MINPP1
PGAM1
LDHA
ALDH3B1
TPI1
PKM
ALDH1A3
ALDOC
GPI
ACSS2
PGK1
Glycolysis / Gluconeogenesis
AK4
RRM2
PAICS
PDE4D
PDE7B
NPR2
PDE3B
GUCY1A2
POLE
AK7
POLR3H
HDDC3
POLR3D
Purine metabolism
QPRT
NMNAT3
Nicotinate and nicotinamide metabolism
Ctrl1
Ctrl2
Ctrl3
IFN γ1
IFN γ2
IFN γ3
La+IFN γ1
La+IFN γ2
La+IFN γ3
C
MDA-MB-231
4T1
Relative CCL5 mRNA expression fold change
Relative CD274 mRNA expression fold change
Relative CXCL10 mRNA expression fold change
Relative CXCL11 mRNA expression fold change
Relative CCL2 mRNA expression fold change
Ctrl
IFN γ
lacidipine+IFN γ
D
Relative CCL5 mRNA expression fold change
E
Relative CD274 mRNA expression fold change
Ctrl
DOX
1-MT+DOX
La (10 mg kg-1)
La (20 mg kg-1)
La (20 mg kg-1)+DOX
Cisplatin
1-MT+Cisplatin
La (20 mg kg-1)+Cisplatin

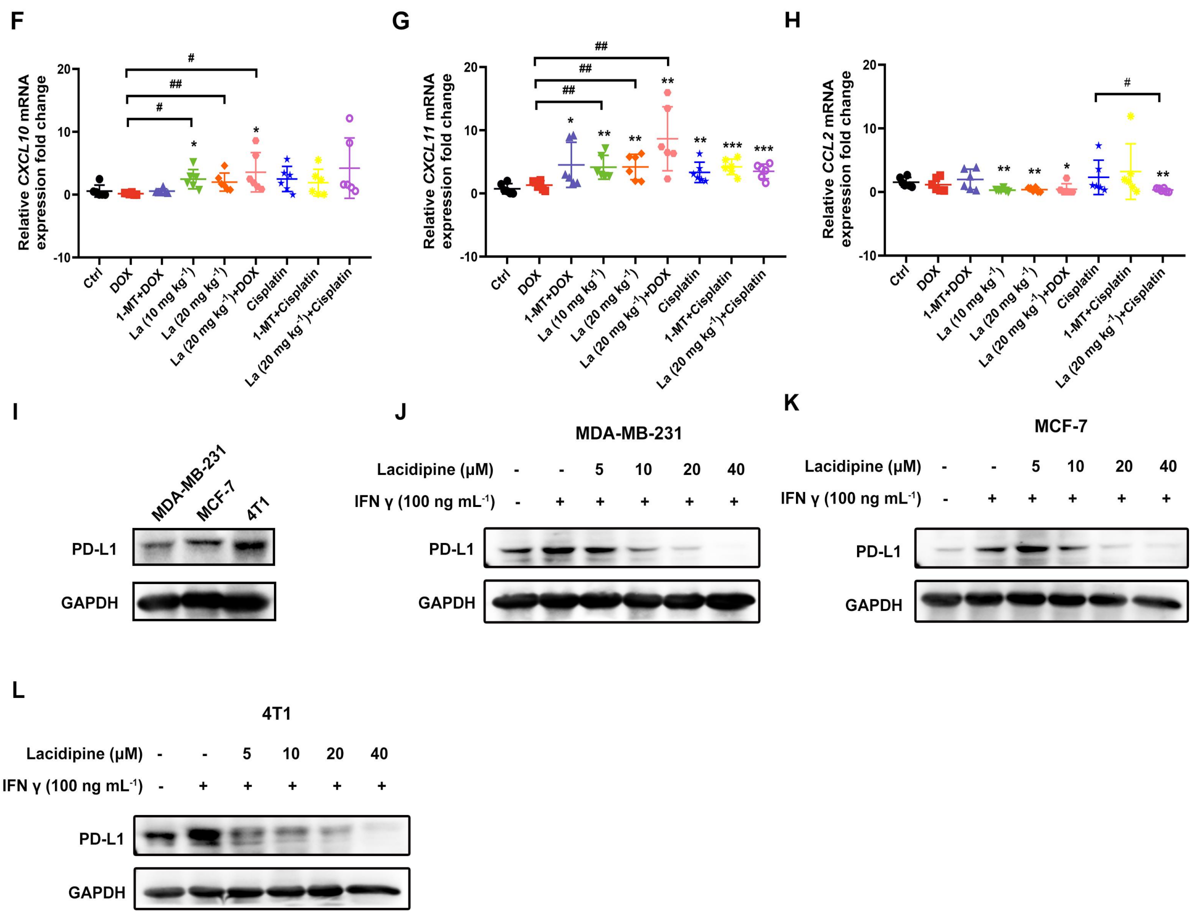

Figure S4. (A) Changes in metabolite levels in the energy metabolic pathway induced by IFN γ alone and in combination with lacidipine (n = 6, each group). *$p < 0.05$, **$p < 0.01$, ***$p < 0.001$ and ****$p < 0.0001$ vs. control group or IFN γ group (unpaired two-tailed Student's *t*-test). (B) Heat map hierarchical clustering displays the differentially expressed genes in the energy metabolism signaling pathway with a p <0.05 (n = 3, each group). (C) MDA-MB-231 and 4T1 cells were treated with lacidipine (20 μM) for 2 h, and then treated with IFN γ (100 ng mL^{-1}) for 24 h. The mRNA expression levels of *CCL5, CD274, CXCL10, CXCL11*, and *CCL2* were examined by qRT-PCR. The mRNA levels of these genes were normalized against the expression level of GAPDH. The experiments were conducted with three independent replicates. Bars, ± SEM. *$p < 0.05$, **$p < 0.01$, and ***$p < 0.001$ vs. the control group and #$p < 0.05$, ##$p < 0.01$ and ###$p < 0.001$ vs. The IFN γ-treated group (unpaired two-tailed Student's *t*-test). (D-H) mRNA expression levels of *CCL5* (D), *CD274* (E), *CXCL10* (F), *CXCL11* (G), and *CCL2* (H) in tumors were examined by qRT-PCR (n = 6, each group). The mRNA levels of these genes were normalized against the expression level of GAPDH. *$p <$ 0.05, **$p < 0.01$, and ***$p < 0.001$ vs. the control group and #$p < 0.05$ and ##$p < 0.01$ vs. the DOX/cisplatin-treated group (unpaired two-tailed Student's *t*-test). La, Lacidipine. (I) Expression of PD-L1 in breast cancer cell lines MDA-MB-231, MCF-7, and 4T1. (J, K, L)

Effect of lacidipine on the PD-L1 expression. MDA-MB-231 (J), MCF-7 (K) and 4T1 (L) cells were pre-treated with lacidipine (5, 10, 20, and 40 μM) for 2 h, then stimulated with IFN γ (100 ng mL^{-1}) for 24 h. The expression of PD-L1 was analyzed by western blotting. GAPDH was used as the loading control.

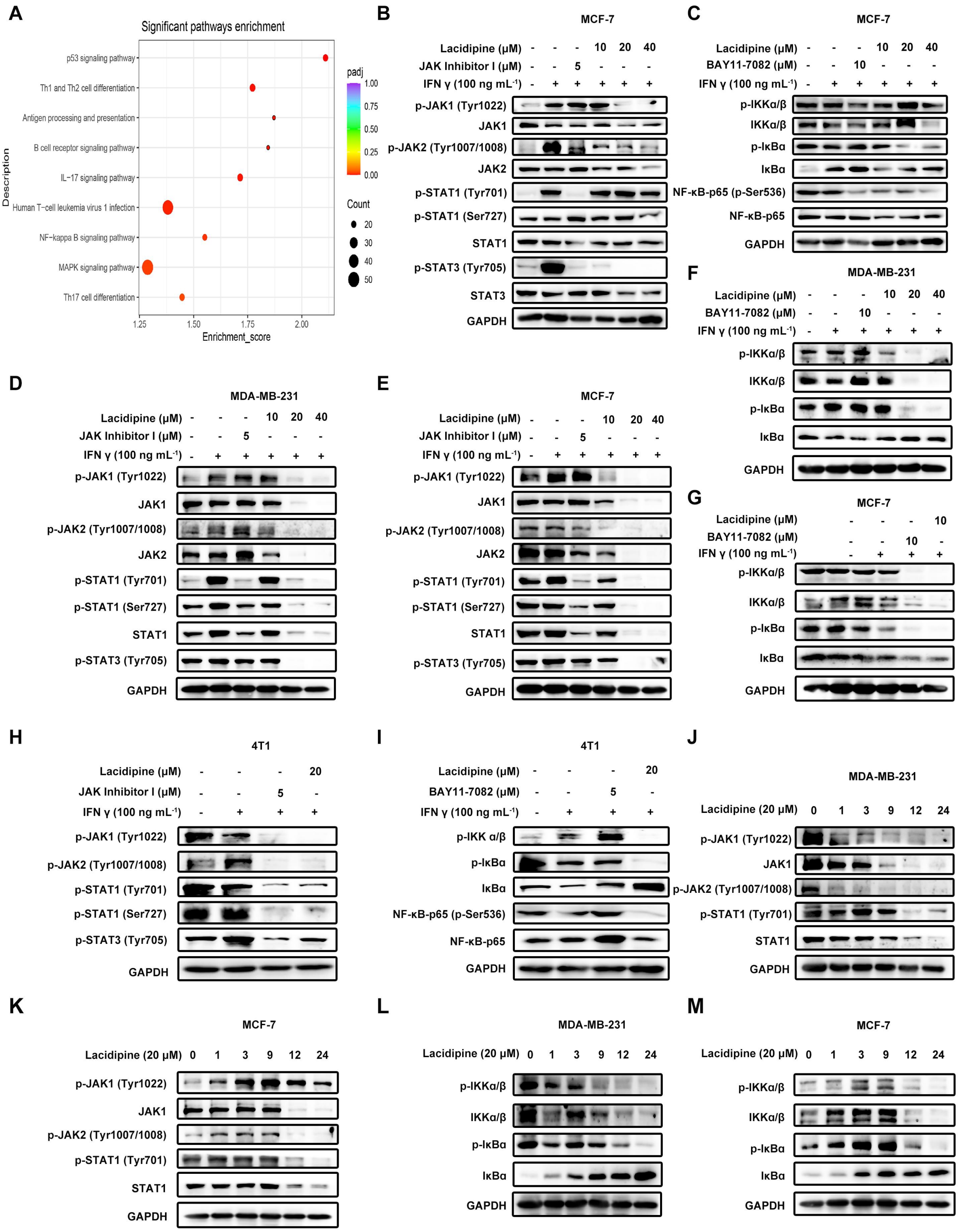

Figure S5. (A) The KEGG pathway enrichment analysis of the top 10 immune-related signaling pathways between IFN γ- and lacidipine + IFN γ-treated groups. (B, C) MCF-7 cells were pre-treated with lacidipine (10, 20, and 40 μM) and JAK inhibitor I (5 μM) or BAY11-7082 (10 μM) for 2 h, then stimulated with IFN γ (100 ng mL^{-1}) for 12 h. The expression of key proteins of the JAK/STAT (B) or NF-κB (C) signaling pathway was analyzed by western

blotting. GAPDH was used as the loading control. (D, E) MDA-MB-231 (D) and MCF-7 (E) cells were pre-treated with lacidipine (10, 20, and 40 μM) and JAK inhibitor I (5 μM) for 2 h, then stimulated with IFN γ (100 ng mL^{-1}) for 24 h. The expression of key proteins of the JAK/STAT signaling pathway was analyzed by western blotting. GAPDH was used as the loading control. (F, G) MDA-MB-231 (F) and MCF-7 (G) cells were pre-treated with lacidipine (10, 20, and 40 μM) and BAY11-7082 (10 μM) for 2 h, then stimulated with IFN γ (100 ng mL^{-1}) for 24 h. The expression of key proteins of the NF-κB signaling pathway was analyzed by western blotting. GAPDH was used as the loading control. (H, I) 4T1 cells were pre-treated with lacidipine (20 μM) and JAK inhibitor I (5 μM) or BAY11-7082 (10 μM) for 2 h, then stimulated with IFN γ (100 ng mL^{-1}) for 24 h. The expression of key proteins of the JAK/STAT (H) or NF-κB (I) signaling pathway was analyzed by western blotting. GAPDH was used as the loading control. (J, K) MDA-MB-231 (J) and MCF-7 (K) cells were treated with lacidipine (20 μM) at various times, and the expression of key proteins of the JAK/STAT signaling pathway was analyzed by western blotting. GAPDH was used as the loading control. (L, M) MDA-MB-231 (L) and MCF-7 (M) cells were treated with lacidipine (20 μM) at various times, and the expression of key proteins of the NF-κB signaling pathway was analyzed by western blotting. GAPDH was used as the loading control. All western blot experiments were conducted with three independent replicates.

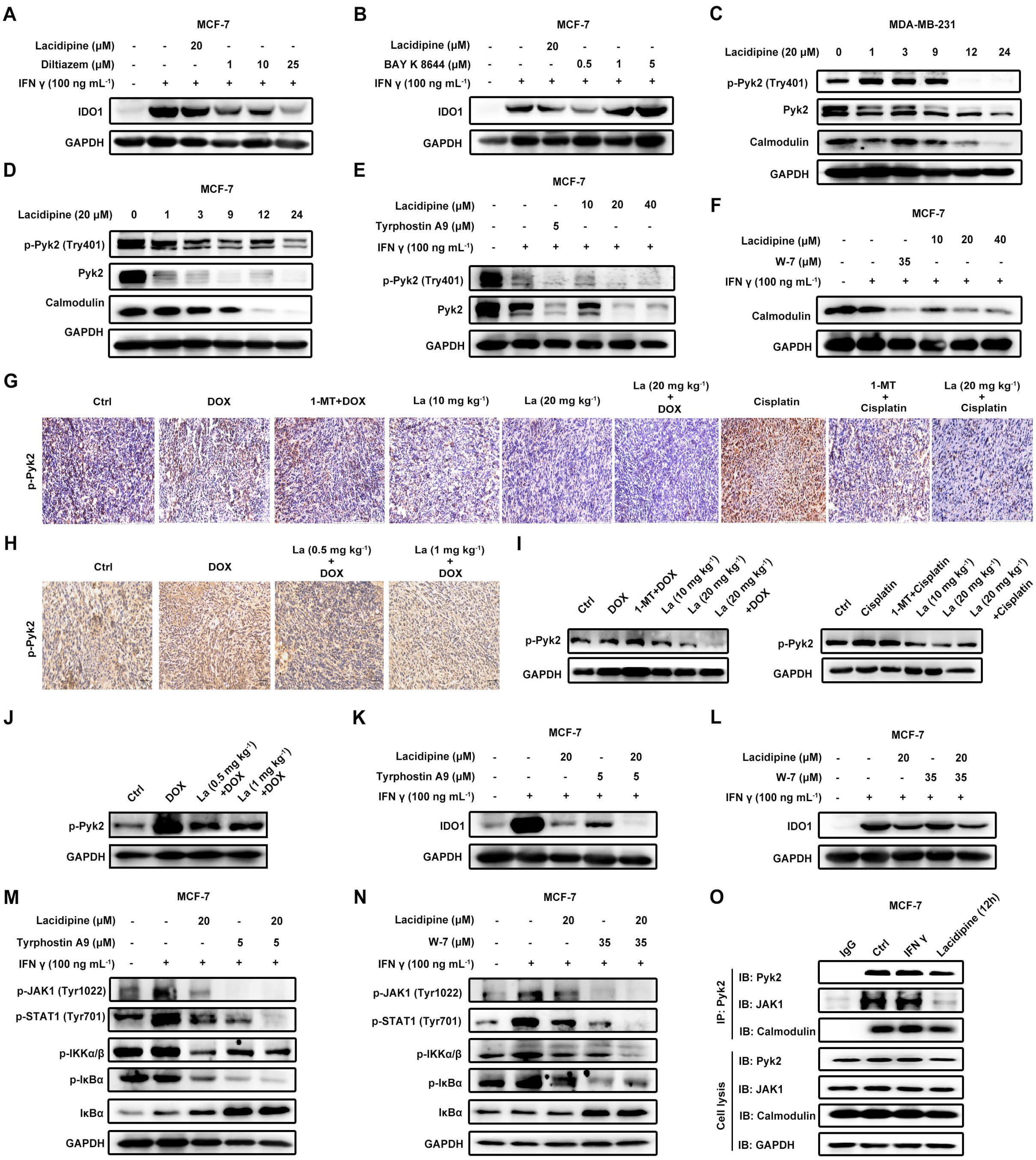

Figure S6. (A, B) MCF-7 cells were pre-treated with lacidipine (20 μM) and diltiazem (1, 10, 25 μM) (A) or BAY K 8644 (0.5, 1, 5 μM) (B) for 2 h, then stimulated with IFN γ (100 ng mL^{-1}) for 24 h. The expression of IDO1 proteins was analyzed by western blotting. GAPDH was used as the loading control. (C, D) MDA-MB-231 (C) and MCF-7 (D) cells were treated with lacidipine (20 μM) at various times, and the expression of Pyk2 phosphorylated at Tyr402, Pyk2, and calmodulin was analyzed by western blotting. (E, F) MCF-7 cells were pre-treated with lacidipine (10, 20, 40 μM) and tyrphostin A9 (5 μM) (E) or W-7 (35 μM) (F) for 2 h, then stimulated with IFN γ (100 ng mL^{-1}) for 24 h. The expression of Pyk2 phosphorylated at Tyr402, Pyk2 and calmodulin proteins was analyzed by western blotting.

GAPDH was used as the loading control. (G, H) The expression of Pyk2 phosphorylaiton was examined in tumor tissues by immunohistochemical analysis (*n* = 6, each group). Representative images are shown. (I, J) The expression of Pyk2 phosphorylation were examined in the homogenate of mice tumor tissues at the end of treatment by western blotting. GAPDH was used as the loading control. (K, L) MCF-7 cells were pre-treated with lacidipine (20 μM) and tyrphostin A9 (5 μM) (K) and W-7 (35 μM) (L) for 2 h, then stimulated with IFN γ (100 ng mL^{-1}) for 24 h. The expression of IDO1 proteins was analyzed by western blotting. GAPDH was used as the loading control. (M, N) MCF-7 cells were pre-treated with lacidipine (20 μM) and tyrphostin A9 (5 μM) (M) and W-7 (N) for 2 h, then stimulated with IFN γ (100 ng mL^{-1}) for 24 h. The expression of phosphorylated JAK at Tyr1022, phosphorylated STAT1 at Tyr701, phosphorylated IKKα/β, phosphorylated IκBα, and IκBα proteins was analyzed by western blotting. GAPDH was used as the loading control. (O) MCF-7 cells were treated with lacidipine (20 μM) for 12 h and then co-immunoprecipitated with Pyk2 antibodies or control immunoglobulin G (IgG) and analyzed for antibody-specific JAK1, calmodulin, Pyk2. All western blot experiments were conducted with three independent replicates.

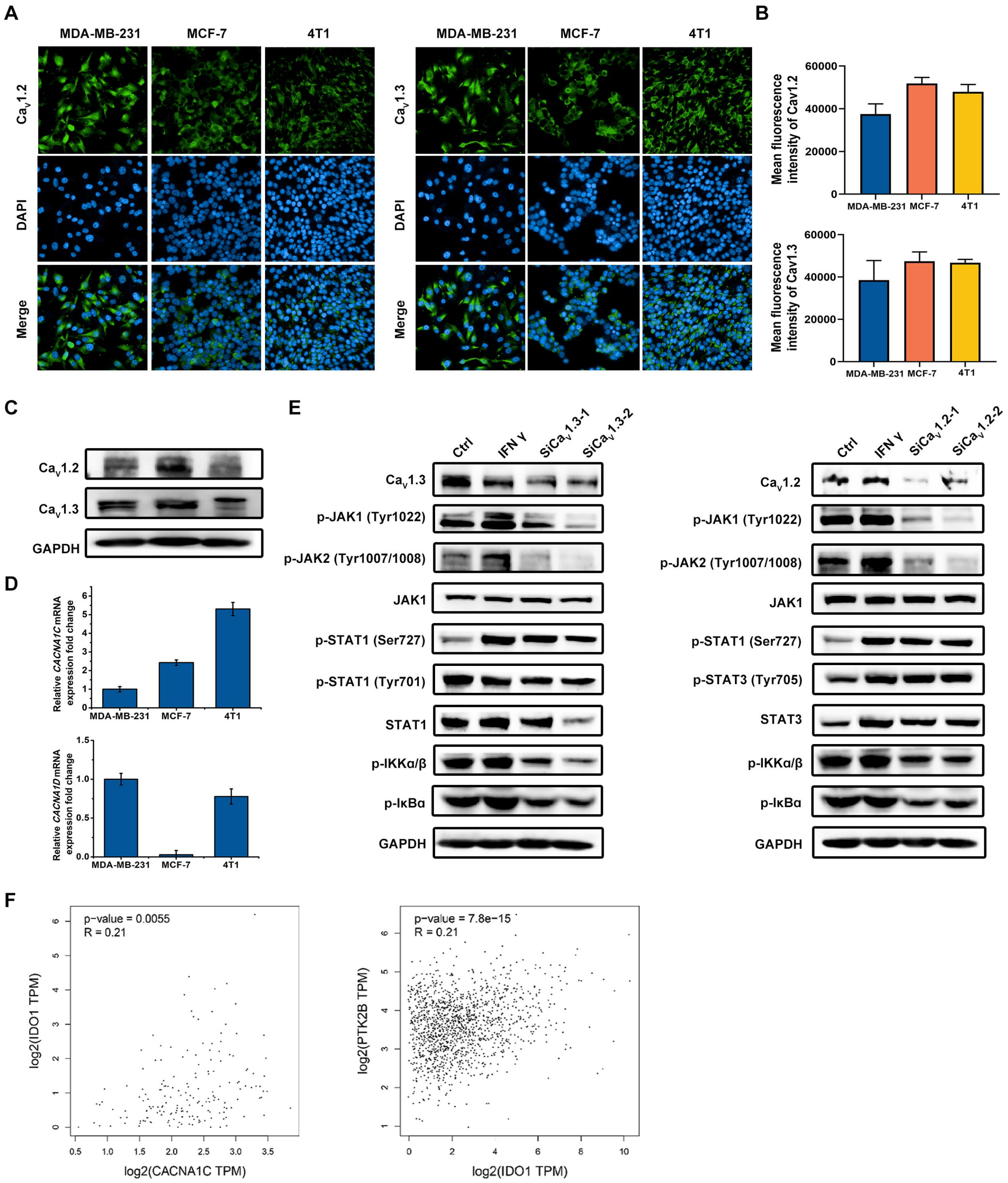

Figure S7. (A) Expression of CaV1.2 and CaV1.3 was analyzed in various cells using a confocal microscope. Scale bar = 100 μm. (B) The mean fluorescence intensity of CaV1.2 and CaV1.3 was analyzed in various cells. (C) The expression of CaV1.2 and CaV1.3 proteins was analyzed by western blotting in various cells. GAPDH was used as the loading control. (D) mRNA expression levels of *CACNA1C* and *CACNA1D* were examined by qRT-PCR in MDA-MB-231, MCF-7, and 4T1 cells. The mRNA levels of these genes were normalized against the expression level of GAPDH. Bars, ± SEM. (E) The related proteins expression

levels of the JAK/STAT and NF-κB signaling pathways were measured after knockdown of $Ca_V1.2$ and $Ca_V1.3$ in MDA-MB-231 cells. All experiments were conducted with three independent replicates. (F) The correlation of $Ca_V1.2$ and IDO1, IDO1 and Pyk2.
